# Exploring the oncostatin M (OSM) feed-forward signaling of glioblastoma via STAT3 in pan-cancer analysis

**DOI:** 10.1186/s12935-021-02260-9

**Published:** 2021-10-26

**Authors:** Miao Chen, Ruiyang Ren, Weimin Lin, Lisha Xiang, Zhihe Zhao, Bin Shao

**Affiliations:** 1 State Key Laboratory of Oral Diseases and National Clinical Research Center for Oral Diseases, West China Hospital of Stomatology, Sichuan University, Chengdu, China; 2 Clinical Trial Center (CTC), NMPA Key Laboratory for Clinical Research and Evaluation of Innovative Drug, West China Hospital, Sichuan University, Chengdu, China; 3 Department of Orthodontics, West China Hospital of Stomatology, Sichuan University, Chengdu, China

## Abstract

**Background:**

Oncostatin M (OSM) has been reported to be a key regulating factor in the process of tumor development. Previous studies have demonstrated both the promotion and inhibition effects of OSM in tumors, therefore inspiring controversies. However, no systematic assessment of OSM across various cancers is available, and the mechanisms behind OSM-related cancer progression remain to be elucidated.

**Methods:**

Based on The Cancer Genome Atlas (TCGA) and Genotype-Tissue Expression (GTEx) databases, we conducted a pan-cancer analysis on OSM to explore its tumor-related functions across cancers as well as its correlations with specific molecules, cells in the tumor microenvironment. Considering the results of pan-cancer analysis, we chose the specific tumor glioblastoma multiforme (GBM) to screen out the OSM-induced signaling pathways and intercellular communications in tumor progression. Wound scratch assay, invasion assay and qRT-PCR were performed to verify the biological effects of OSM on glioblastoma cells.

**Results:**

Higher OSM level was found in most tumor tissues compared with corresponding normal tissues, and the enhanced OSM expression was observed to be strongly related to patients’ poor prognosis in several cancers. Moreover, the expression of OSM was associated with stromal and immune cell infiltration in the tumor microenvironment, and OSM-related immune checkpoint and chemokine co-expression were also observed. Our results suggested that OSM could communicate extensively with the tumor microenvironment. Taking GBM as an example, our study found that two critical signaling pathways in OSM-related tumor progression by KEGG enrichment analysis: Jak-STAT and NF-κB pathways. Single-cell RNA sequencing data analysis of GBM revealed that OSM was mainly secreted by microglia, and cell–cell interaction analysis proved that OSM-OSMR is an important pathway for OSM to stimulate malignant cells. In vitro, OSM treatment could facilitate the migration and invasion of glioblastoma cells, meanwhile promote the proneural-mesenchymal transition. The administration of STAT3 inhibitors effectively suppressed the OSM-mediated biological effects, which proved the key role of STAT3 in OSM signaling.

**Conclusion:**

Taken together, our study provides a comprehensive understanding with regard to the tumor progression under the regulation of OSM. OSM seems to be closely related to chronic inflammation and tumor development in the tumor microenvironment. As an important inflammatory factor in the tumor microenvironment, OSM may serve as a potential immunotherapeutic target for cancer treatment, especially for GBM.

**Supplementary Information:**

The online version contains supplementary material available at 10.1186/s12935-021-02260-9.

## Background

Oncostatin M (OSM), a member of the IL-6 family, has been considered an important cytokine in diverse physiological and pathological processes. Previous studies have found its contributions in both innate and adaptive immunity, hematopoiesis, homeostasis, and osteogenesis [1, 2]. And its roles in autoimmunity and inflammation are also widely reported [3, 4]. Our previous study indicated that OSM was highly expressed in jaw bone marrow, serving as an important inflammatory factor that regulated the homeostasis of jaw bone [5]. In particular, OSM presents pleiotropic functions on cancers. Firstly, its inhibitory effects are confirmed on several tumors like melanoma, fibrosarcoma, rhabdomyosarcoma, ovarian, lung, stomach, and breast carcinoma [6]. Some studies deeply demonstrated that the inhibitory effects of OSM in cancers were mainly associated with the deceleration of tumor cell proliferation and the promotion of cell differentiation rather than cell apoptosis or cell death [7, 8]. However, more studies further reported its synergistic effect with tumorigenesis, epithelial-mesenchymal transformation (EMT), angiogenesis, metastasis, and chemoresistance [9–11]. Although a large number of studies describe its specific roles across various cancers, there is still a lack of systematic studies to determine the role of OSM across various cancer types.

Mature OSM consists of 196 amino acids [12], which is mainly secreted by macrophages and neutrophils [13, 14]. Though OSM can be secreted in normal physiological activities, inflammation is the major situation for its secretion [15, 16], which is closely related to tumor development. OSM-mediated downstream signal transduction depends on two specific heterodimers on the cell surface: gp130/OSM receptor (OSMR) and gp130/leukemia inhibitory factor receptor (LIFR). OSM possesses the widest range of downstream signaling pathways among the IL-6 family [2, 17], to name a few, Jak-STAT3, ERK1/ERK2, phosphatidylinositol-3-kinase (PI3K)/Akt, and NF-κB pathways [18–21]. STAT3 is the most widely reported OSM downstream signaling pathway, which is also considered to be an important node connecting inflammation and cancer. In the tumor microenvironment, STAT3 not only up-regulates the expression of genes related to proliferation, survival, invasion and metastasis, but also promotes the production of a variety of cytokines, chemokines and other mediators (such as interleukin-6 and cyclooxygenase-2), and these mediators are involved in inflammation-related tumorigenesis. In addition, these factors will further promote the activation of STAT3 and form a paracrine or autocrine-dependent feedback loop, leading to a continuous increase of inflammation in the tumor microenvironment [22].

Although OSM was initially considered a tumor-inhibiting factor by in vitro experiment [23], recent lines of evidence confirmed its roles to promote tumor development as well as reduce survival time of patients suffering from cancers [9, 24]. As a result, the functions and mechanisms of OSM across cancers remain to be elucidated. To systematically explore the regulatory role of OSM across various cancer types, we explored the public available databases to implement a pan-cancer analysis of OSM. We discussed the relationship between OSM expression and survival prognosis, immune cell infiltration, immune checkpoints and cytokines, DNA repair genes and methyltransferases in 33 cancers, and further explored through gene enrichment analysis and single-cell RNA sequencing (scRNA-seq) analysis. Based on the results of bioinformatics analysis, we explored the biological effects and underlying mechanism of OSM on glioblastoma cells.

## Methods and materials

### OSM expression analysis in pan-cancer

We collected data of tumor cell lines in the Cancer Cell Line Encyclopedia (CCLE) database and normal tissue data in Genotype-Tissue Expression (GTEx) database for our OSM gene expression analysis. We further utilized the data in The Cancer Genome Atlas (TCGA) and the corresponding normal tissue data in GTEx database to estimate the expression discrepancy between tumor and normal tissues. RNA sequencing and clinical data of 33 cancer types in TCGA database were as follows:

Adrenocortical carcinoma (ACC), bladder urothelial carcinoma (BLCA), breast invasive carcinoma (BRCA), cervical squamous cell carcinoma (CESC), cholangiocarcinoma (CHOL), colon adenocarcinoma (COAD), lymphoid neoplasm diffuse large B cell lymphoma (DLBC), esophageal carcinoma (ESCA), glioblastoma multiforme (GBM), brain lower grade glioma (LGG), head and neck squamous cell carcinoma (HNSC), kidney chromophobe (KICH), kidney renal clear cell carcinoma (KIRC); kidney renal papillary cell carcinoma (KIRP), acute myeloid leukemia (LAML), liver hepatocellular carcinoma (LIHC), lung adenocarcinoma (LUAD), lung squamous cell carcinoma (LUSC), mesothelioma (MESO); ovarian serous cystadenocarcinoma (OV), pancreatic adenocarcinoma (PAAD), pheochromocytoma and paraganglioma (PCPG), prostate adenocarcinoma (PRAD), rectum adenocarcinoma (READ), sarcoma (SARC), skin cutaneous melanoma (SKCM), stomach adenocarcinoma (STAD), testicular germ cell tumors (TGCT), thyroid carcinoma (THCA), thymoma (THYM), uterine corpus endometrial carcinoma (UCEC), uterine carcinosarcoma (UCS), and uveal melanoma (UVM). All the OSM expression data were converted to the log2 [TPM (Transcripts per million) + 1] forms, which were applied to draw violin plots.

The expression and distribution of OSMR protein across pan-cancer were visualized via the human protein atlas database (https://www.proteinatlas.org/). The proportion of OSMR expression intensity in different tumor tissues was counted.

### Survival analysis

We chose three indicators (OS: overall survival, DFI: disease-free interval, and PFI: progression-free interval) to describe the survival time and prognosis of patients suffering from various cancers by using the clinical information in TCGA database. Forest plots were shown to estimate the correlation between OSM level and patient prognosis. And data of specific cancers with statistical significance were further presented in Kaplan–Meier curves for survival analysis.

### Cell infiltration analysis

We applied ESTIMATE (Estimation of STromal and Immune cells in MAlignant Tumor tissues using Expression data) algorithm [25] to explore the correlation between cell infiltration status (stromal and immune cells) and OSM level in the tumor microenvironment. Spearman’s rank correlation test was utilized to calculate *p* values as well as partial correlation values. The results were presented in the way of scatter plots. Furthermore, we chose OSM highly-relevant cancers to conduct OSM density distribution analysis, presented by density distribution maps. TIMER (Tumor Immune Estimation Resource; https://cistrome.shinyapps.io/timer) algorithm [26] was used to estimate specific immune cell infiltration status including dendritic cell, macrophage, neutrophil, B cell, CD4^+^ T cell, and CD8^+^ T cell, and their relationship with OSM. The data were visualized as scatter plots.

### Co-expression analysis

We conducted Pearson’s correlation analysis between OSM and the expressions of four specific clusters of molecules (immune checkpoints, chemokines, growth factors and cytokines). Four correlation heatmaps were created, and each described the OSM-related co-expression status of one type of these molecules. *p* values and correlation values were visualized in hierarchical colors. The abnormality of tumor DNA mismatch repair (MMR) and epigenetic modification is closely related to tumor progression. With the online analysis tool sanger box (http://sangerbox.com/), we explored the relationship between DNA MMR genes, DNA methyltransferase (DNMTs) and OSM expression across multiple-typed cancers.

### Different gene expression and gene enrichment analysis

Firstly, we applied the STRING tool (https://string-db.org/), setting “OSM” as the target to produce a PPI network to visualize the main OSM-binding proteins for subsequent pathway analysis. According to the expression level of OSM, we divided GBM patients in TCGA into two groups with OSM high expression and OSM low expression, and analyzed differential genes through the limma package [27]. Subsequently, we used a website-based tool metascape (http://metascape.org/gp/index.html) [28] to perform KEGG and GO enrichment analysis of pathways in GBM. The main pathways which showed strong correlations with OSM levels were visualized in grouped networks. We also sorted these pathways by *p* values and listed them in histograms. To conduct GSEA analysis [29], we used the GO gene sets and KEGG gene sets, which were downloaded from http://www.gsea-msigdb.org/gsea/downloads.jsp. The related pathways were selected for visualization in R software.

### Single-cell RNA sequencing analysis of GBM tumor

ScRNA-seq data of GBM samples were downloaded from publicly published data of four human primary samples [30]. Seurat package [31] was used for the reanalysis of scRNA-seq data. The ratio of mitochondria < 25% and 100 < nFeature_RNA < 6000 were set as thresholds to filter cells. Uniform manifold approximation and projection (UMAP) algorithm was utilized for data dimension reduction. A scatter diagram was presented to describe the characteristic proteins of different cells. To identify the cell interaction and specific pathways involved in the GBM tumor microenvironment, we used the cellphonedb2 package [32]. Cell–cell interactions were shown through heatmap and network plot. The cytokine-related interaction pathways between microglia and other cell types were selected and displayed.

### Cell culture

Human glioblastoma cell line U251 was purchased from the China Center for Type Culture Collection (CCTCC). The U251 cells were cultured in DMEM/F12 medium, supplemented with 10% Fetal Bovine Serum (FBS) and 1% penicillin–streptomycin in a 5% CO_2_ humidified incubator at 37 °C. The culture medium was changed every two days, and cells were passaged through trypsin digestion at 80% confluence.

### Cell proliferation assay

U251 cells were seeded in a 96-well plate at a density of 5000 cells per well, and OSM were added at concentration of 0, 10, 50 ng/mL. After culturing for 1 day, 3 days, and 5 days, 10 μl of CCK8 (Dojindo Laboratories) reagent was added to each well and then placed in the incubator to continue incubating for 1 h, and the absorbance at 450 nm was measured with a microplate reader.

### Scratch assay

Glioblastoma cells U251 were seeded in 6-well plates and cultivated to 80% confluence. After 12 h of culture in serum-free medium, the cells in each plate were scratched with a 200 μL pipette tip. Then the cells were treated with 10 ng/mL OSM (Abnova), 50 ng/mL OSM or 50 ng/mL OSM + 6 μg/mL cryptotanshinone (STAT3 inhibitor, Meilunbio) respectively in serum-free medium, and observed in 0, 24, or 72 h time points using randomly selected microscopic view under an inverted microscope at 200 × magnification. The wound healing rate was calculated through ImageJ software.

### Invasion assay

For cell invasion assay, the matrigel invasion chamber was rehydrated with serum-free medium at 37 °C for 2 h. Cell incubation was conducted with OSM and cryptotanshinone in serum-free DMEM. The inner chamber was added with cell suspension, while the outer chamber consisted of 15% FBS DMEM media. After 24 or 72 h of incubation, 4% PFA was applied for cell fixation, and 0.2% crystal violet for staining. After staining, the chamber was observed under an inverted microscope. The numbers of migrating cells were counted under the microscope at 200× magnification. Statistical results of migrating cell numbers from three independent experiments were averaged from six image fields.

### Quantitative reverse transcription polymerase chain reaction (qRT-PCR)

Total RNA of cultured cells was extracted using Trizol reagent (Invitrogen), and then reversely transcribed to obtain stable cDNA using PrimeScript™ RT reagent Kit with gDNA Eraser (TaKaRa Bio). The qRT-PCR was performed using SYBR Premix Ex Taq II (TaKaRa Bio) in Quant Studio™ 3 real-time fluorescent quantitative PCR instrument (ThermoFisher Scientific). Glyceraldehyde 3-phosphate dehydrogenase (GAPDH) was used as an internal reference to normalize the gene expression [29]. The result was calculated using the 2^−ΔΔCt^ method and expressed as a multiple change relative to GAPDH.

### Western blot

After OSM and STAT3 inhibitor treatment, the total proteins were extracted by RIPA buffer (Pierce, Rockford, IL) on ice. Equal quantities of protein samples were separated by electrophoresis on 12% SDS-PAGE polyacrylamide gels. Then, the samples were electro-transferred to PVDF membranes (0.22 μm, Millipore) using a wet transfer apparatus (Bio‐Rad) and blocked with 5% BSA in PBS for 1 h at room temperature. The membranes were incubated overnight at 4 °C with primary antibodies of CD44, FN1, CHI3L1, CD24, DLL3, OLIG2 and β-ACTIN (Abcam), respectively. After that, the blots were incubated with horseradish peroxidase (HRP)-conjugated secondary antibodies (#HA1001, huabio, 1:5000) at room temperature for 1 h [30]. The immobilon reagents (Millipore) was used for the visualization and detection of antibody-antigen complexes. The band intensity was measured by ImageJ software.

### Chromatin immunoprecipitation sequencing (ChIP-seq) analysis

To analyze the binding site of STAT3 in human genome, we downloaded the STAT3 ChIP-seq data from the gene expression omnibus (GEO) database (GSE31477) [33] and analyzed it on an online website tool CistromeDB (http://cistrome.org/db/#/). The binding peaks of STAT3 in the OSMR, LIFR, and IL6R gene ranges were visualized and marked in red frames.

### Statistical analysis

All quantified data are expressed as mean ± standard deviation (SD). Statistical differences were performed via student’s t test or one-way analysis of variance (ANOVA) followed by the Tukey’s post hoc test for comparisons. *p* values < 0.05 were considered to be statistically significant.

## Results

### OSM expression in normal and tumor tissues across pan-cancer

**Fig. 1:**
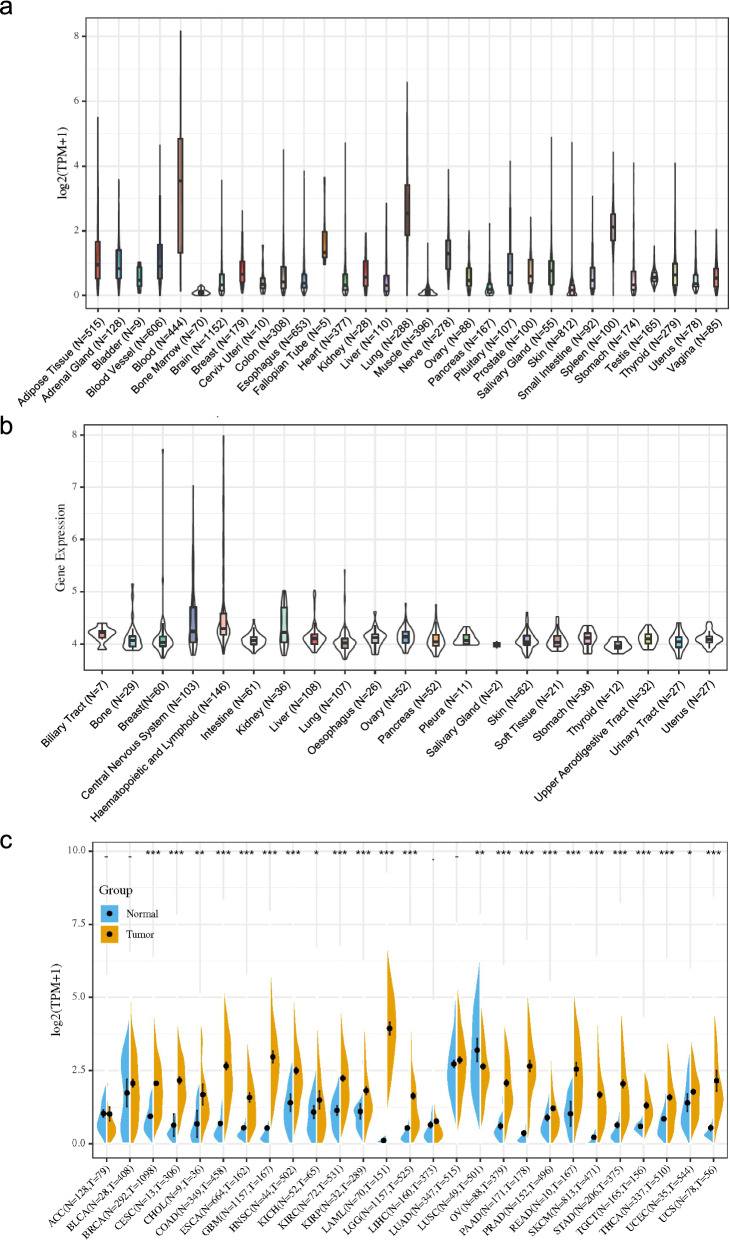
OSM expression in cancer tissues and normal tissues. **a** Violin plots of OSM expression data in normal tissue in GTEx database. **b** Violin plots of OSM expression data in cancer cell lines in CCLE database. **c** Combined analysis of OSM expression in GTEx and TCGA database. *p < 0.05, **p < 0.01, ***p < 0.001

Our study aims to probe into the expression of OSM in normal controls and tumor tissues across multiple cancer types. Firstly, we described the mRNA expression of OSM in normal tissues in GTEx database. As illustrated in Fig. 1a, OSM gene is commonly expressed in various normal tissues including epithelial tissues, connective tissues, muscle, and nervous system. More specifically, OSM gene shows the highest transcription level in the blood, followed by lung and spleen (Fig. 1a). Kruskal–Wallis test showed an obvious difference in OSM expression among tissues. Furthermore, we downloaded the data of each tumor cell line from the CCLE database, and analyzed the OSM expression level in 21 tissues which were divided according to the tissue source (Fig. 1b). Similarly, OSM was highly expressed in tumor cell lines of blood, lung and spleen.

To understand the discrepancy of OSM expression between tumors and normal tissues, we subsequently performed a comparison of OSM expression across 20 cancers in TCGA database (Additional file 1: Fig. S1). Considering that there were insufficient normal samples in TCGA, we integrated the normal tissue data in the GTEx and tumor tissue data in TCGA to analyze the OSM expression differences among 27 tumors (Fig. 1c). The result indicated that most of the detected cancers (23 out of 27 tumor tissues), including BRCA, CESC, COAD, ESCA, GBM, HNSC, KIRC, KIRP, LAML, LGG, OV, PAAD, PRAD, READ, SKCM, STAD, TGCT, THCA, UCS (P < 0.001), CHOL, LUSC (*p* < 0.01), KICH and UCEC (*p* < 0.05) expressed OSM with higher levels compared with the corresponding normal tissues. Only in LUSC we have detected a lower expression of OSM in malignant tissues which was statistically significant (*p* < 0.01). Using immunohistochemical staining data in human protein atlas, we evaluated the expression of OSM receptors (OSMRs) in 20 types of cancer. The expression of OSMR could be detected across all cancer types, among which urothelial cancer expressed the highest, while the expression level in prostate cancer was relatively low (Additional file 1: Fig. S2).

### Survival analysis of OSM in pan-cancer

**Fig. 2:**
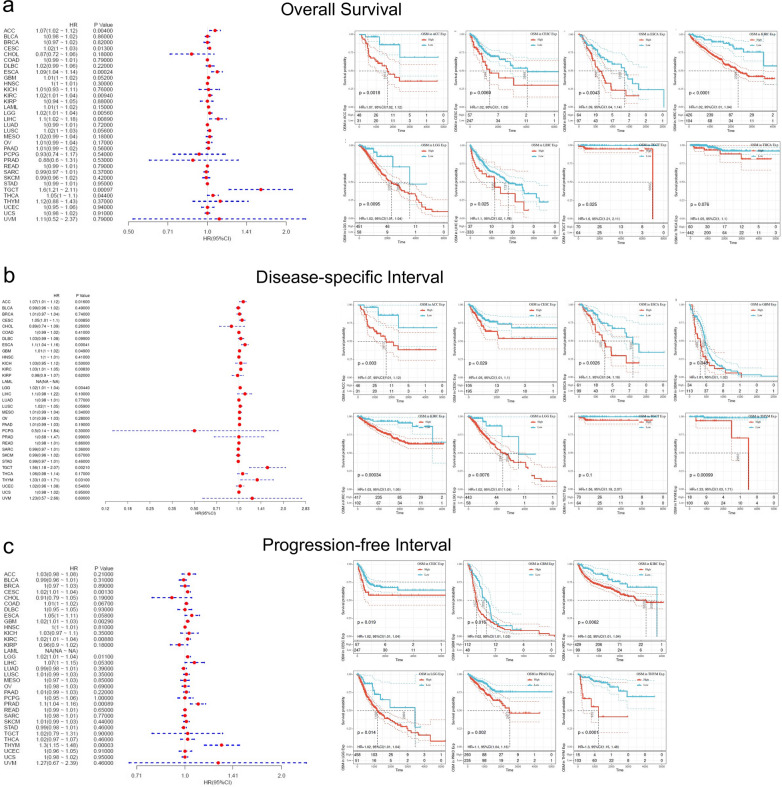
Correlation analysis between OSM level and overall survival, disease-specific survival, and progression-free interval in cancer patients based on TCGA database. **a** Forest plot to describe OSM-related overall survival of cancer patients and Kaplan–Meier curves for overall survival analysis in ACC, CESC, ESCA, KIRC, LGG, LIHC, TGCT, and THCA. **b** Forest plot to describe OSM-related disease-specific survival of cancer patients and Kaplan–Meier curves for disease-specific survival analysis in ACC, CESC, ESCA, GBM, KIRC, LGG, TGCT, and THYM, and THCA. **c** Forest plot to describe OSM-related progression-free interval of cancer patients and Kaplan–Meier curves for progression-free interval analysis in CESC, GBM, KIRC, LGG, PRAD, and THYM. Hazard ratios and *p* values are shown

To explore the influence of high OSM expression in the prognosis of cancer patients, we divided TCGA patients into OSM high expression group and low expression group to conduct analysis of OS, DFI, and PFI. For OS analysis, in patients of ACC (p = 0.004), ESCA (p = 0.00024), KIRC (p = 0.0094), LGG (p = 0.0056), LIHC (p = 0.0089), and TGCT (p = 0.00097), the higher OSM levels could result in poorer overall survival (OS) (Fig. 2a), among which the TGCT group showed the largest difference of patients’ OS with a distinct level of OSM in the tumor microenvironment (HR = 1.6 with 95% CI of 1.21–2.11). As detailed in Fig. 2a, the overall survival rate of all the eight cancer types differed significantly in low and high OSM groups. For instance, in ACC patients, the 50% OS rate of the high OSM group appeared in 1613 days after the cancer diagnosis, while in the other group 50% of patients survived more than 5000 days.

According to the Cox regression analysis, ACC (*p* = 0.016), CESC (*p* = 0.0085), ESCA (*p* = 0.00041), GBM (*p* = 0.049), KIRC (*p* = 0.0083), LGG (*p* = 0.0044), TGCT (*p* = 0.0021) and THYM (*p* = 0.031) were observed that higher expression levels of OSM were risk factors of patients’ poorer disease-specific survival (Fig. 2b). Taking these cancer data to form survival curves, the diagrams plotted apparent survival advantages in most patients with cancer tissues of lower OSM expression degree (Fig. 2b).

Likewise, the hazard ratio analysis of PFI had similar results that five cancer types have presented a significantly higher risk for tumor progression with high OSM expression. Among these five cancers, CESC (*p* = 0.0013), KIRC (*p* = 0.0088), LGG (*p* = 0.011), PRAD (*p* = 0.00089), and THYM (*p* < 0.0001) respectively, THYM demonstrated the largest discrepancy between two groups, showing that half the number of controlled THYM with low OSM expression underwent at least five times as long to have further deterioration than OSM-abundant ones (Fig. 2c).

Collectively, our survival analysis revealed that the higher level of OSM seemed to be a risk factor for the survival of various cancer types. According to the results of OS, DFI and PFI analysis, we presented strong evidence that in nerve system tumors (LGG and GBM), higher OSM expression showed a high positive correlation with both patients’ mortality and malignant progression. Interestingly, there were no evidence of any cancer type, hinted that high expression of OSM was related to a better prognosis.

### OSM-related cell infiltration analysis in pan-cancer

**Fig. 3:**
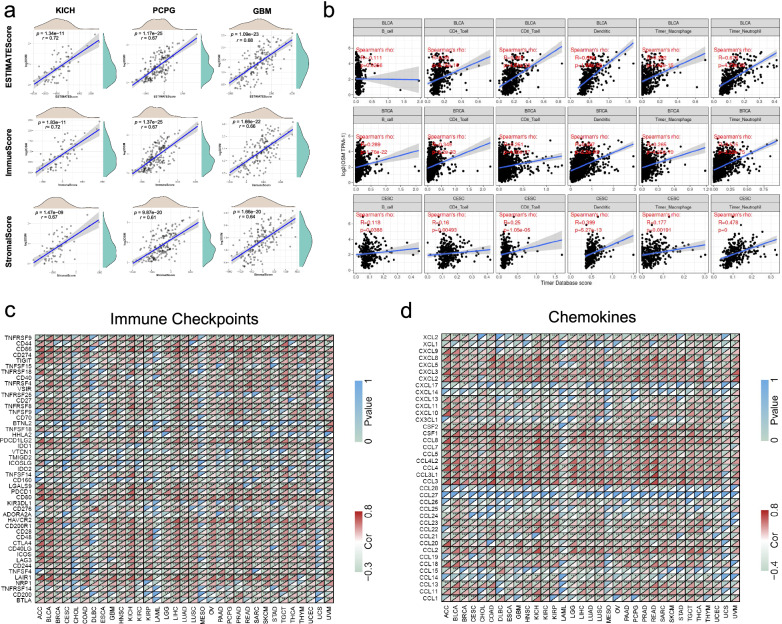
OSM-related cell infiltration and molecule expressions in tumor microenvironment. **a** OSM-related ESTIMATE score, immune score and stromal score in tumor microenvironments of the top3 cancer types (KICH, PCPG and GBM) via ESTIMATE algorithm. Spearman’s *p* values and partial correlation values are displayed. And density distribution of OSM levels, stromal scores, immune Scores, and ESTIMATE scores are also plotted. **b** Correlation analysis between OSM expression and the specific immune cell types in BLCA, BRCA, and CESC via TIMER algorithm. Dendritic cell, macrophage, neutrophil, B cell, CD4^+^ T cell, and CD8^+^ T cell are involved. Spearman’s *p* values and partial correlation values are displayed. **c** Correlation heatmap of OSM-related immune checkpoints across 33 cancer types in TCGA. **d** Correlation heatmap of OSM-related chemokines across 33 cancer types in TCGA. The colors in the bottom right corner of each grid represent Spearman’s correlation values. And the colors in the upper left corner of each grid represent Spearman’s *p* values. *p < 0.05, **p < 0.01, ***p < 0.001

Immune regulation of microenvironment plays an important role through the whole process of tumor development, and considerable pieces of evidence show that the progression of tumor closely correlates with chronic inflammation [34]. As a cytokine, the rise of OSM in concentration will probably induce cytokine crosstalk and mediate immune responses in the tumor microenvironment via various signal pathways, among which STAT3 is known as the major downstream molecule so far [35]. Based on ESTIMATE algorithm, we brought in “ESTIMATE Score”, “Stromal Score”, and “Immune Score” to evaluate stromal cell infiltration, immune cell infiltration and tumor purity in the tumor microenvironment, respectively (Additional file 1: Fig. S3–S5). According to ESTIMATE analysis, OSM levels in most malignant tissues were positively related to the stromal and immune infiltration degree, and negatively related to tumor purity. Both Stromal and Immune Scores of BRCA, COAD, and GBM showed high correlations with regard to OSM, while in terms of tumor purity, the ESTIMATE Scores of BRCA, CESC, and COAD demonstrated strong associations with OSM. The results suggested that OSM may function as an activator for both immune cell infiltration and stromal cell proliferation. Further, we utilized the top three cancer types that possessed the strongest association with OSM expression to perform density distribution analysis with regard to OSM level. We noticed that OSM expression levels in these cancers were approximately skewed distributions, and most cancer tissues were infiltrated in the environment with a relatively low concentration of OSM (Fig. 3a).

Besides the overall estimation of immune cell infiltration, we were also interested in the correlation between OSM expression and the specific immune cell type. To address this issue, we explored the correlations between OSM gene expression and immune infiltration level of diverse immune cells, which contained innate immune cells (dendritic cell, macrophage, and neutrophil) and adaptive immune cells (B cell, CD4^+^ T cell, and CD8^+^ T cell). We applied TIMER algorithms to measure the enrichment of immune cells. As illustrated by Fig. 3b, the majority of immune cells in these three cancers were positively related to the enhancement of OSM expression (Fig. 3b). Specifically, higher enrichments of dendritic cells in BLCA (R = 0.689, *p* = 4.91e−28), neutrophils in BRCA (R = 0.515, *p* = 3.22e−75) and neutrophils in CESC (R = 0.478, *p* = 0) were observed the strongest correlation with OSM increase, respectively.

### OSM-related co-expression in pan-cancer

OSM has been proven to be an inflammatory factor with multiple regulatory effects, but the relationship between OSM and the expression of some important genes in the tumor microenvironment has rarely been reported. Here, we explored the correlation between OSM expression and immune checkpoints, growth factors, cytokines and chemokines through Pearson’s correlation analysis in 33 cancers. It was worth noting that, as shown in Fig. 3c, most of the immune checkpoints had a strong positive correlation with OSM expression, among which CD86, LAIR1, HAVCR2 and PDCD1LG2 were significantly positively associated with OSM expression in most cancer types. Chemokines, as the main component of the tumor inflammatory microenvironment, play an important role in tumor growth, metastasis and immune evasion [36–38]. It seemed that a large number of chemokines were observed to co-express with OSM to a large extent, especially CCL3/3L1/4/4L2/5/7/8, CSF1 as well as CXCL2/3/8 (Fig. 3d). The top three growth factors with strong correlations with OSM in most cancers were HGF, PDGFB, and TGFB1. However, the correlations between the expressions of OSM and typical growth factors across various cancer types were relatively weak, while the growth factors that regulate angiogenesis exhibited a stronger correlation with OSM (Additional file 1: Fig. S6a). By contrast, the expression of most inflammatory factors had a closer correlation with OSM expression (Additional file 1: Fig. S6b).

**Fig. 4:**
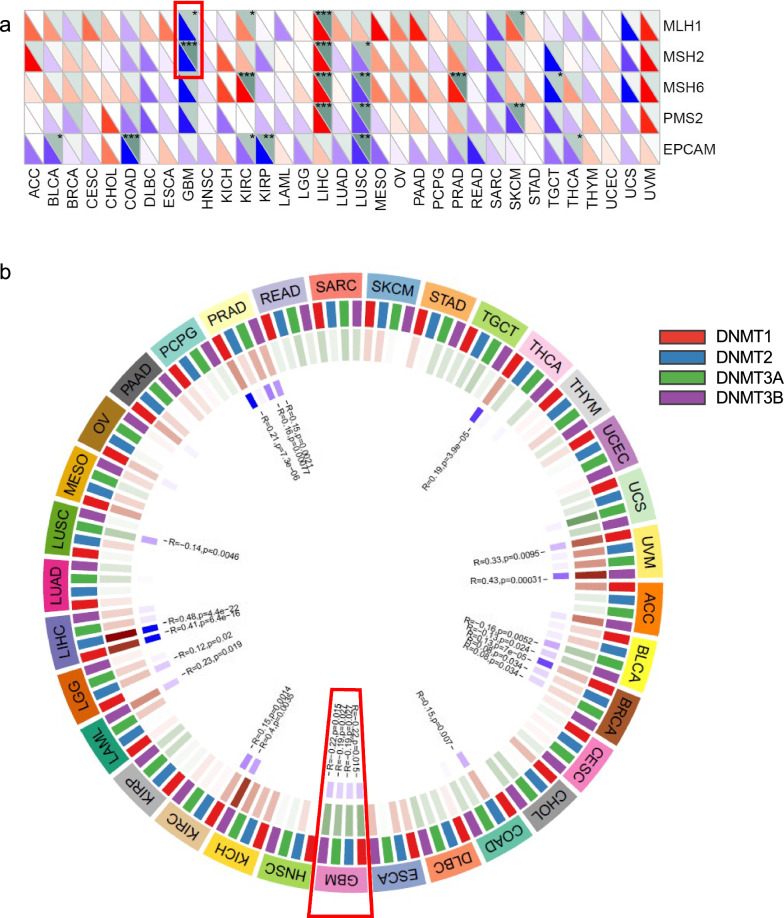
Correlation analysis between OSM expression and MMR gene, DNA methyltransferase gene expressions. **a** Heatmap of OSM-related five MMR gene co-expression (MLH1, MSH2, MSH6, PMS2, and EPCAM) of different cancers in TCGA. The colors in the bottom left corner of each grid represent Spearman’s correlation values. And the colors in the upper right corner of each grid represent Spearman’s *p* values. **b** Visualization of OSM-DNA methyltransferase gene co-expression. The colors of red, blue, green, and purple represent DNMT1, DNMT2, DNMT3A, and DNMT3B, respectively

Moreover, we analyzed the correlation between OSM gene expression and the expression of four DNA methyltransferases (DNMT1: red, DNMT2: blue, DNMT3A: green, and DNMT3B: purple). In GBM, the OSM expression showed a correlation with the expression of all these four methyltransferases. And three methyltransferases were found correlations in PRAD and BRCA (Fig. 4b). Two out of five MMR genes in GBM, namely MLH1 and MSH2, as plotted in Fig. 4a, showed a significant negative correlation with the expression level of OSM, indicating a high somatic mutation risk when exposed in high OSM environment.

### Different gene expression and pathway enrichment in GBM patients

**Fig. 5:**
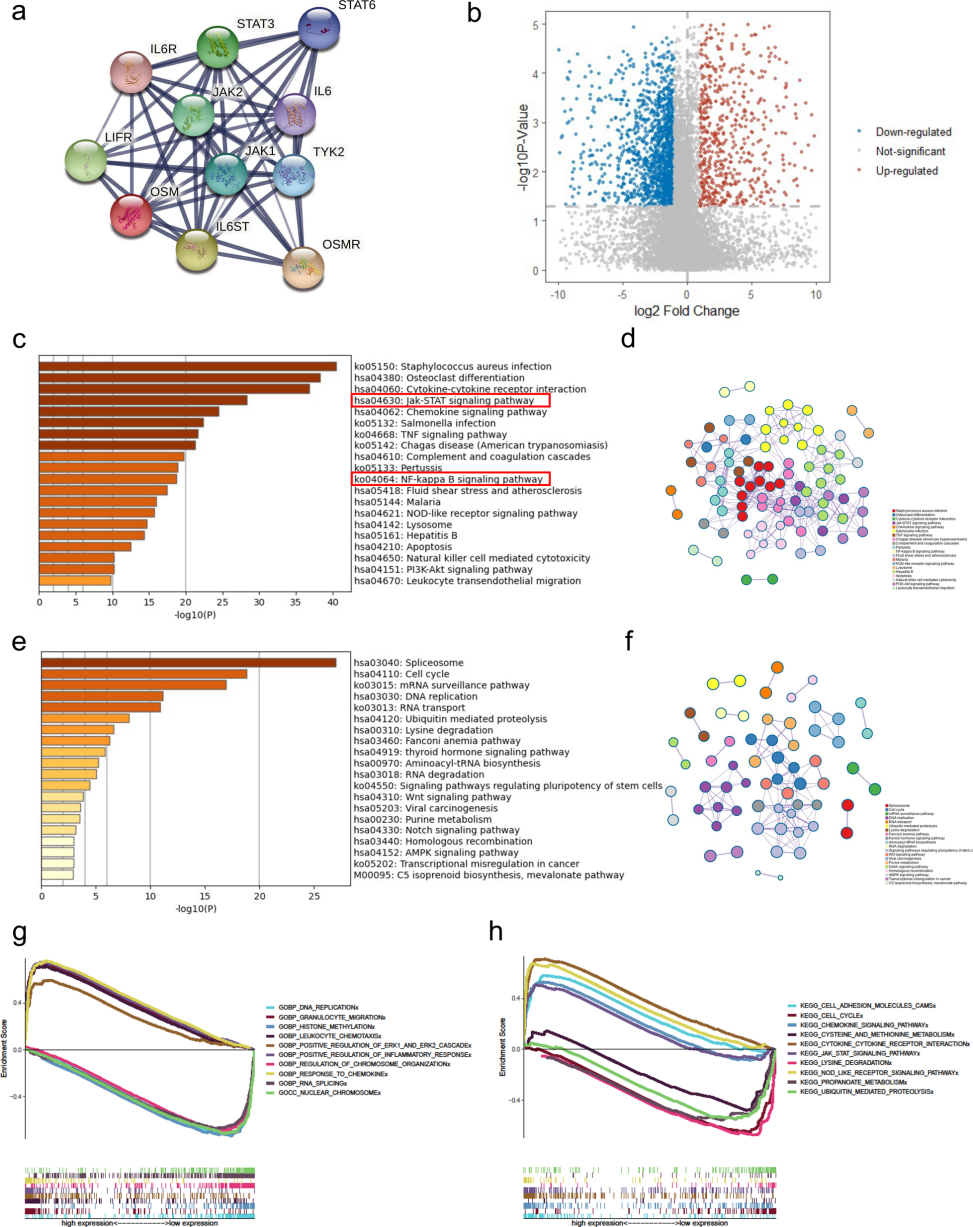
Main proteins and signaling pathways with regard to OSM-induced tumor progression. **a** PPI network of main OSM-binding proteins via STRING analysis. **b** Volcano plot shows the distribution of OSM-up-regulated and down-regulated genes. **c** Histogram of main OSM-up-regulated pathways in the OSM high expression group based on KEGG enrichment analysis. **d** Network plot to visualize the enriched pathways. The number of circles with identical colors represents the enrichment degree of each pathway. **e** Histogram of main OSM-down-regulated pathways in the OSM high expression group based on KEGG enrichment analysis. **f** Network plot to visualize the enriched pathways. **g** Enrichment plot of the main OSM-up-regulated and down-regulated pathways via GSEA analysis of GO gene sets. **h** Enrichment plot of the main OSM-up-regulated and down-regulated pathways via GSEA analysis of KEGG gene sets

GBM is one of the most dangerous brain cancers with high mortality [39]. Our findings above suggested that the composition of GBM microenvironment had a strong correlation with OSM expression, therefore, we chose GBM to further analyze the mechanisms of OSM in tumor development. Via STRING analysis, we demonstrated 10 important OSM-binding proteins presented in PPI network, which suggested that OSM can bind to several receptors (OSMR, LIFR, and IL6R) and activate downstream signaling transduction (Jak1/2, STAT3/6, and TYK2) (Fig. 5a). To screen out specific pathways by which OSM may contribute to tumor progression, we divided GBM patients in TCGA into OSM high expression group and OSM low expression group according to the expression of OSM. Through differential gene analysis, we found that compared with the low expression group, 744 genes in the OSM high expression group were up-regulated and 1480 genes were down-regulated (*padj* < 0.001, |logFC| > 1) (Fig. 5b). To further explore the functions of these differential genes, we implemented KEGG and GO enrichment analysis. Our KEGG enrichment analysis indicated that two cancer-promoting pathways, “Jak-STAT pathway” and “NF-κB pathway”, might be the probable approaches for OSM to induce cancer progression, which were in line with the PPI network. While at the same time, the GO enrichment showed that the immune and inflammatory responses (especially the leukocyte activation and migration) were significantly up-regulated by OSM (Fig. 5c, d, Additional file 1: Fig. S7a, b). The down-regulated activities by OSM were mainly generated at DNA or RNA level, and the negative impact on cell cycle was another important regulating mechanism (Fig. 5e and f, Additional file 1: Fig. S7c and d). Similarly, GESA analysis also supported that immune cell activation and inflammatory response-related gene sets were up-regulated in OSM high expression group (Fig. 5g, h).

### Glioblastoma scRNA-seq reveals OSM-related gene expression and its regulatory effect

**Fig. 6:**
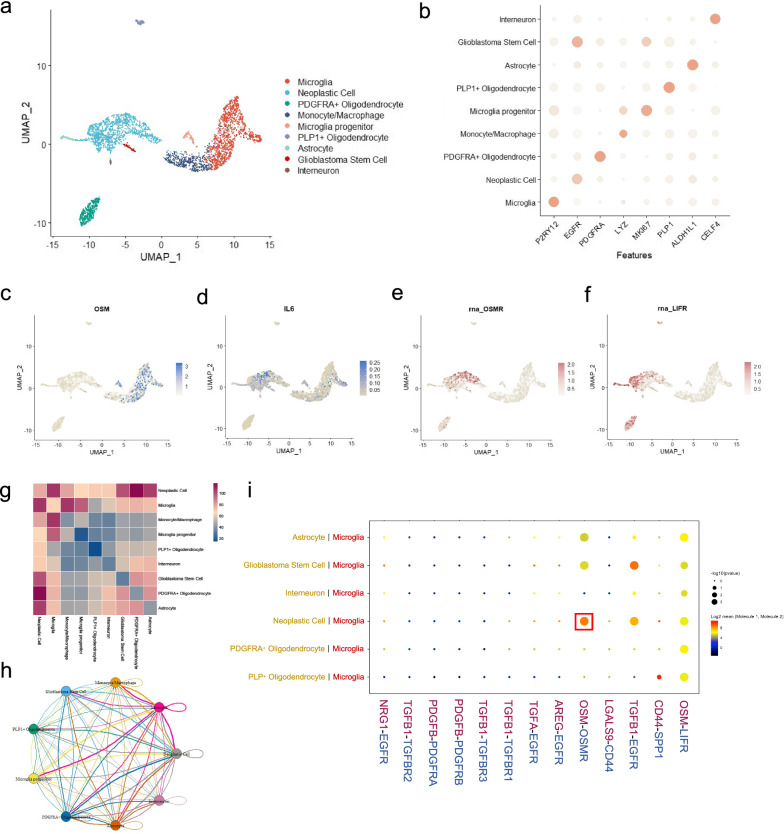
Expression of OSM-related molecules and cell–cell interactions in glioblastoma scRNA-seq data. **a** Identification of the various cell types in GBM tumor microenvironment. **b** Scatter diagram of the characteristic gene expression of different cell types. **c**–**f** Visualization of the distribution of ligand OSM/IL6 and receptor OSM/LIFR expression in various cell types in GBM tumor microenvironment. **g** Heatmap to describe the intercellular communication frequency of various cell types. The color of each grid represents the cell–cell interaction score. **h** Network plot of intercellular communications among selected cell types. **i** Visualization of the interaction pathways between microglia and other cell types. The size of the circle represents *p* value. The color of the circle demonstrates the mean value of cell–cell interaction

To further explore the source and target of OSM in the GBM tumor microenvironment, we analyzed the human GBM scRNA-seq data in a public database. Through several reported markers, we have identified that non-immune cells mainly included GBM neoplastic cells, GBM stem cells, oligodendrocytes, astrocytes, and interneurons. Immune cells mainly included microglia and monocytes/macrophages (Fig. 6a, b). In GBM microenvironment, OSM was mainly expressed by microglia population. While the most important corresponding receptors, OSMR and LIFR, were mainly expressed in GBM neoplastic cells and PDGFRA^+^ oligodendrocytes. IL-6 was found in both tumor cells and microglia (Fig. 6c–f). Subsequently, we conducted a cell–cell interaction study via cellphonedb2 package to evaluate the ligand-receptor interactions between different cell populations in GBM microenvironment. From the heatmap and network plots in Fig. 6g, h, we could speculate that neoplastic cells kept in close contact with neighboring astrocytes, PDGFRA^+^ oligodendrocytes, tumor stem cells as well as microglia. To further understand the mechanisms of communications across various cell types in GBM, several signal pathways that had been reported to be essential for tumor progression were taken into account. Notably, six cell populations in GBM microenvironment (astrocyte, GBM stem cell, interneuron, neoplastic cell, PDGFRA^+^ oligodendrocyte, and PLP1^+^ oligodendrocyte) interacted with microglia by one shared pathway: OSM-LIFR pathway. Astrocytes, GBM stem cells, and neoplastic cells were also closely connected with microglia via another pathway: OSM-OSMR. Two types of malignant cell types, neoplastic cells and GBM stem cells, utilized TGFβ1-EGFR pathway to implement strong communication with microglia as well (Fig. 6i). These results suggested that the microglia served as an important regulator of neoplastic cells in GBM microenvironment, and the OSM signal pathway was an important approach for tumor-microglia interaction.

### OSM promotes the invasion and proneural-mesenchymal transition of glioblastoma cells

**Fig. 7:**
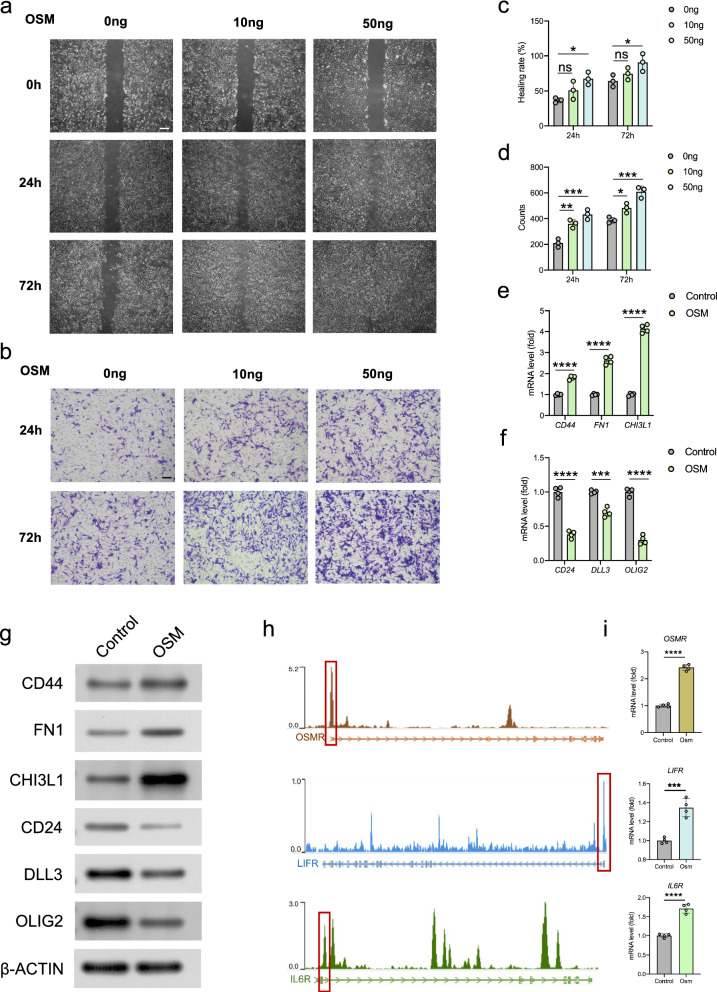
Role of OSM in migration, invasion and proneural-mesenchymal transition of glioblastoma cells. **a** The wound healing of glioblastoma cells using scratch assay. Scale bar, 50 μm. Magnification, 200×. **b** Statistical analysis of wound healing rates. **c** Invasive potential of glioblastoma cells in matrigel invasion assay. Scale bar, 50 μm. Magnification, 200×. **d** Statistical analysis of the number of cell counts in each field of view. **e** mRNA levels of mesenchymal signature genes in control and OSM-treated group. **f** mRNA levels of proneural signature genes in control and OSM-treated group. **g** Representative immunoblots showing levels of CD44, FN1, CHI3L1, CD24, DLL3 and OLIG2 in U251 cells. β-ACTIN was the loading control. **h** ChIP-seq analysis of the binding site of STAT3 to OSMR, LIFR and IL6R. **i** mRNA levels of OSM-related receptors (OSMR, LIFR and IL6R) in control and OSM-treated group. *p < 0.05, **p < 0.01, ***p < 0.001, ****p < 0.0001

We first explored the effect of OSM on the proliferation of glioblastoma cells in vitro. However, OSM treatment has no significant effect on cell proliferation at both low and high concentrations (Additional file 1: Fig. S8a). The migration and invasion characteristics are two significant indicators to measure the aggressiveness of tumor cells [40]. The invasion and migration potential of GBM can be mediated by various proteins like integrins and cadherins, as well as interactions of different cells and molecules in the tumor microenvironment [41]. In this study, we observed the migration potential of glioblastoma cells in both control and OSM-treated cell groups by scratch assay. The migration and invasion potential of glioblastoma cells enhanced with the increase of OSM concentration (Fig. 7a–d). Further, to investigate OSM-related proneural-mesenchymal transition in glioblastoma cells, we observed the changes of proneuronal signature genes and mesenchymal signature genes after OSM treatment, and found that the increase of three mesenchymal signature genes, CD44, FN1, and CHI3L1, were statistically significant, while three proneuronal signature genes, CD24, DLL3, and OLIG2, experienced obvious declines (Fig. 7e, f). Through western blot, we found that the expression of mesenchymal signature genes (CD44, FN1, and CHI3L1) increased while the expression of proneuronal signature genes (CD24, DLL3, and OLIG2) decreased (Fig. 7g). STAT3 is considered to be the most important signal molecule downstream of OSM and can act as a transcription factor to activate the transcription of target genes. The analysis of ChIP-seq data of STAT3 proved that STAT3 could bind to the transcription initiation region of three OSM-specific receptors, OSMR and LIFR, as well as the most common receptor in IL6 family (IL6R). In addition, the expressions of these three receptor genes were all experienced obvious increases under OSM-stimulation (Fig. 7g, h).

### The biological effects of OSM to glioblastoma cells are STAT3-dependent

**Fig. 8:**
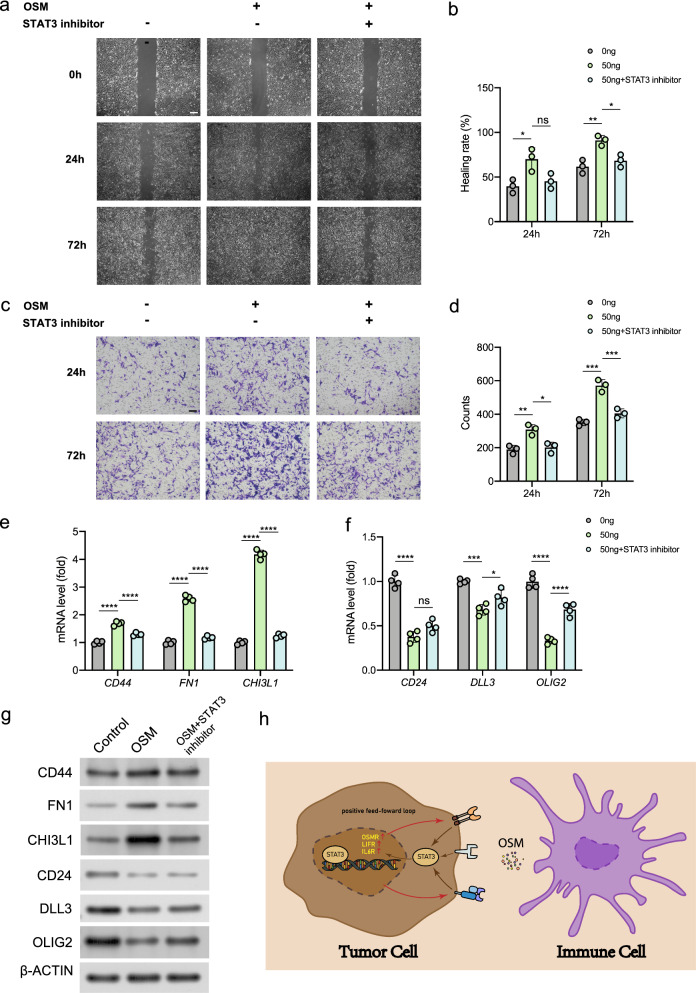
Impact of STAT3 signaling pathway in OSM-induced glioblastoma cell migration, invasion and proneural-mesenchymal transition. **a** The wound healing of OSM-treated glioblastoma cells before and after inhibition of STAT3. Scale bar, 50 μm. Magnification, 200×. **b** Statistical analysis of wound healing rates. **c** Invasive potential of glioblastoma cells in matrigel invasion assay. Scale bar, 50 μm. Magnification, 200×. **d** Statistical analysis of the number of cell counts in each field of view. **e** mRNA levels of mesenchymal signature genes in control, OSM-treated, and STAT3-inhibited groups. **f** mRNA levels of proneural signature genes in three groups. **g** Representative immunoblots showing levels of CD44, FN1, CHI3L1, CD24, DLL3 and OLIG2 in U251 cells. β-ACTIN was the loading control. **h** Graphical illustration of the OSM positive feed-forward loop in tumor cell. *p < 0.05, **p < 0.01, ***p < 0.001, ****p < 0.0001

To verify the key role of STAT3 in the biological effects of OSM, we used cryptotanshinone, a STAT3 inhibitor, to block the function of STAT3 under OSM treatment. Disturbed by STAT3 inhibitor, the healing rate of glioblastoma cells demonstrated a significant decline after 72 h of culture (Fig. 8a, b). Marked by cell counts, the invasion potential after STAT3 pathway interruption decreased in 24 h and 72 h (Fig. 8c, d). qRT-PCR results demonstrated that the inhibition of STAT3 strongly suppressed the increase of mesenchymal signature genes induced by OSM, while partially reversed the inhibitory effect of OSM on proneural signature genes (Fig. 8e, f). Also, by western blot, we validated the expression changes of the mesenchymal and proneuronal signature genes at protein level (Fig. 8g, Additional file 1: Fig. S8b). Taken together, these results suggested that OSM could act as a stimulator of GBM proneural-mesenchymal transition process as well as migration and invasion potential, which functioned by activation of STAT3 (Fig. 8h, Additional file 1: Fig. S8c).

## Discussion

OSM has been continuously studied based on several other tumors with similar verdicts since the first discovery of its ability to confront A375 melanoma by inhibiting cell proliferation [23, 42, 43]. Recent researches, on the contrary, elucidate that the overexpression of OSM is closely related to the promotion of tumor progression, including cell proliferation, immunoregulation, angiogenesis, and metastasis [35]. However, the role of OSM across various cancers remains to be elucidated in more detail. In this study, we have estimated the functions of OSM, the correlation between OSM expression and the cell enrichment in the tumor microenvironment as well as the interactions among kinds of cells. Based on TCGA and GTEx, our findings hint that most tumor tissues are equipped with higher levels of OSM mRNA expression compared to the corresponding normal tissue, which indicates the activation of the OSM gene in the most tumor microenvironments and potentially demonstrates the positive regulation of OSM in tumor progression. To gain further insight into the OSM-related conditions of patients suffering from cancers, a series of survival analyses (OS, DFI, DSS, and PFI) was implemented. Our results evidently present that high OSM level is correlated with poor patient prognosis in several cancers including ACC, CESC, ESCA, GBM, KIRC, LGG, LIHC, PRAD, TGCT, THCA, and THYM, which is in line with the previous studies [24, 44].

Growing evidence points to that the status of tumors is closely related to the composition and infiltrating concentration of cells in their corresponding environment, and that the component ratio and purity of cell types in tumor tissues tie tightly with intercellular communication [45–47]. In the present study, the stromal scores and immune scores across most cancers increase with the rising of OSM levels. Some studies have confirmed that OSM functions as an important activator of stromal cells. As a member of the IL-6 family, OSM can cause higher responses from stromal cells than other IL-6 members since OSM receptors express at a higher level compared with IL-6 receptors [48]. Immune cell infiltration analysis indicates that OSM is proportionate to the concentration of most immune cell types in BLCA, BRCA and CESC. Since OSM is secreted mostly by myeloid immune cells, we can infer that the high expression of OSM is a consequence of tumor-related inflammation. Previous studies have revealed the interaction between tumorigenesis and inflammations [34, 49–51]. In turn, in the tumor microenvironment, OSM has been reported to positively regulate EMT [52], degradation of ECM [53] and cell-substrate detachment of tumor cells [54], thus leading to metastatic transformation. According to our correlation analysis between OSM expression and specific immune cell infiltration, we make a point that the OSM-induced chronic inflammation may be mainly contributed by macrophages and neutrophils [4, 55–57]. The regulations of various cells including tumor cells by OSM are extensively studied [58–60], however, enough shreds of evidence are still needed to demonstrate the relationship between OSM and diverse immune cells and the mechanisms.

Interestingly, although the expression of OSM is positively correlated with the abundant immune cell infiltration inside the tumor, prognostic analysis proves that OSM usually leads to poor clinical outcomes, indicating the potential immune cell dysfunction in anti-tumor response. Immune checkpoint co-expression analysis indicates the possibility of OSM to activate immune checkpoints in the tumor inflammatory environment, which can lead to immune exhaustion and tumor progression despite a large number of immune cell infiltration. Moreover, the up-regulated chemokines that accompany OSM may also serve as manipulation targets to regulate tumor progression. Abnormal changes in the tumor genome regulation are closely related to the occurrence and development of tumors. DNA mismatch repair is a highly-conserved approach to maintain genome stability and is executed in three steps: mismatch recognition, excision and DNA re-synthesis [61]. The dysfunction of key genes in the process will cause the failure of DNA repair, leading to a higher probability of somatic mutations. At the epigenetic level, abnormal expression of key tumor genes can be also caused by abnormal genomic methylation in tumors, which can be reminded by changes in DNA methyltransferase (DNMT) [62, 63]. Our analysis on DNA methylation and MMR indicates that the cancer-promoting effects of OSM are related to both abnormal DNA methylation and high-frequency somatic mutation, especially in GBM.

Since previous studies and our present result confirmed an important role of OSM in GBM [64–67], we have focused on this particular tumor type, exploring intercellular communication based on scRNA-seq data. GBM neoplastic cells interact with most types of cells in high frequencies, suggesting that glioblastoma cells might play a central role in regulating the surroundings for a more suitable environment. Moreover, the cell distribution statuses of OSM/OSM-receptors hint that the neoplastic cell-microglia predominate in OSM-based intercellular communications. In addition, our findings also confirm the OSM signal pathways again on cell–cell interaction, where OSM-LIFR and OSM-OSMR are two major cytokine-cytokine receptor interactions in GBM-related cell communications. Gene enrichment analysis suggests that the downstream pathways Jak-STAT and NF-κB may serve as two important mechanisms for OSM to promote tumor development. Herein, we give a possible explanation inferred from our results. On one hand, as mentioned above, OSM utilizes two signaling pathways, Jak-STAT and NF-κB, which leads to the mesenchymal-type changes in tumor cells and self-feedback enhancement of OSM signaling pathway. On the other hand, although OSM promotes immune cell infiltration, the expressions of immune checkpoints are also increased, leading to dysfunction or functional inhibition of immune cells.

Finally, to verify our above findings based on bioinformatics analysis, we explore the potential biological effects and mechanisms of OSM in the human glioblastoma cell line U251. We find that OSM could effectively promote the migration and invasion ability of glioblastoma cells. According to molecular expression characteristics, GBM can be divided into four subtypes (Proneural, Neural, Classic, and Mesenchymal) [68], among which mesenchymal subtypes show higher aggressiveness and worse prognosis [69, 70]. After OSM treatment, the mesenchymal signature genes of glioblastoma cells are significantly up-regulated while the expression of proneural signature genes decreases. In terms of mechanism, the results of KEGG enrichment analysis suggest that the STAT pathway is activated in patients with high OSM expression. STAT3 is the most important downstream effector of OSM, which has been reported to be continuously activated in the GBM microenvironment and promote tumor invasion and progression [67, 71]. Remarkably, the ChIP-seq data shows STAT3 not only binds to the transcription initiation region of OSMR, but also LIFR and IL6R. Through qRT-PCR, we confirm that OSM treatment could up-regulate the expression of OSMR, LIFR and IL6R, suggesting that there is a wide range of positive feed-forward loops in the OSM regulatory signal network. Mechanistically, we prove that STAT3 signaling plays a key role in OSM-mediated biological effects. The OSM-mediated enhancement of migration and invasion of glioblastoma cells is significantly reduced after STAT3 inhibitor treatment, and the expression of gene markers related to neuron-mesenchymal transition is also restored. The experimental results support the role of OSM in promoting the progression of GBM, in which STAT3 serves as a key downstream signaling molecule.

Collectively, we have explored the correlations between OSM expression and various tumor-related cells and molecules. Subsequently, we focus on GBM to dig deeper insight for OSM-mediated intercellular communications as well as signal pathway activation. Despite being a cytokine that was originally found to inhibit tumor cell proliferation, OSM is found to be associated with the progression of many tumors. The positive feedforward loops mediated by STAT3 play an important role in OSM-mediated tumorigenesis. Further studies are needed to explore the mechanisms behind OSM-related molecule co-expression and immune cell infiltration. Lacking animal experiment to verify the effect of OSM is a major limitation in current study. So far, no effective molecular inhibitor against OSM has been reported. Therefore, OSM knockout mouse may serve as an ideal animal model to explore the effects of OSM on tumor development. Our research suggests that OSM is an important factor in the occurrence and progression of multiple cancer types, and targeted inhibition of OSM may be an effective cancer treatment approach.

## Supplementary Information

**Additional file 1: Fig. S1** OSM expressions in cancer tissues and the corresponding normal tissues based on TCGA database. The OSM mRNA levels across 20 cancer types between cancer tissues and normal tissues in TCGA. * *p* < 0.05, ** *p* < 0.01, *** *p* < 0.001. **Fig. S2** Immunohistochemical staining revealed OSMR expressions in 20 cancer tissues. **a** Stacked bar plot to visualize the degree of OSMR gene expression based on immunohistochemical staining in 20 cancer types. **b** Immunohistochemical staining of OSMR protein in 20 cancers. The patient IDs, genders, ages as well as the cancer types are shown. **Fig. S3** Estimation of OSM-related tumor purity (demonstrated by ESTIMATE Scores) in the tumor microenvironments across cancers in TGCA via ESTIMATE algorithm. Spearman’s *p* values and partial correlation values are displayed. **Fig. S4** Estimation of OSM-related stromal cell infiltration (demonstrated by Stromal Scores) in the tumor microenvironments across cancers in TGCA via ESTIMATE algorithm. Spearman’s *p* values and partial correlation values are displayed. **Fig. S5** Estimation of OSM-related immune cell infiltration (demonstrated by Immune Scores) in the tumor microenvironments across cancers in TGCA via ESTIMATE algorithm. Spearman’s *p* values and partial correlation values are displayed. **Fig. S6** Co-expression analysis of specific molecules (growth factors and cytokines) and OSM of cancers in TCGA. **a** Heatmap of OSM-related growth factor co-expression analysis. **b** Heatmap of OSM-related cytokine co-expression analysis. The colors in the bottom right corner of each grid represent Spearman’s correlation values. And the colors in the upper left corner of each grid represent Spearman’s *p* values. * *p* < 0.05, ** *p* < 0.01, *** *p* < 0.001. **Fig. S7** Main OSM-related signaling pathways in tumor microenvironment via GO enrichment analysis. **a** Histogram of main OSM-up-regulated pathways in the OSM high expression group based on GO enrichment analysis. **b** Grouped network plot to visualize the enriched pathways. The number of circles with identical colors represents the enrichment degree of each pathway. **c** Histogram of main OSM-down-regulated pathways in the OSM high expression group based on GO enrichment analysis. **d** Grouped network plot to visualize the enriched pathways. **Fig. S8 a** The effect of OSM on the proliferation of U251 cells. **b, c** Gray values of bands of CD44, FN1, CHI3L1, CD24, DLL3 and OLIG2.

## Data Availability

The transcriptome data and clinical information of 33 cancer types are downloaded from the TCGA database (https://portal.gdc.cancer.gov/). Human glioblastoma scRNA-seq data can be downloaded from http://www.gbmseq.org/. ChIP-seq data of STAT3 can be downloaded from the GEO database with accession number GSE31477 [33].
