# Supplementary material for: Exploring the oncostatin M (OSM) feed-forward signaling of glioblastoma via STAT3 in pan-cancer analysis
Source: Cancer Cell Int. 2021 Oct 26;21:565. doi: 10.1186/s12935-021-02260-9 (PMC8549168; doi:10.1186/s12935-021-02260-9)
Supplement: Supplementary file 1 — Additional file 1: Fig. S1 OSM expressions in cancer tissues and the corresponding normal tissues based on TCGA database. The OSM mRNA levels across 20 cancer types between cancer tissues and normal tissues in TCGA. * p < 0.05, ** p < 0.01, *** p < 0.001. Fig. S2 Immunohistochemical staining revealed OSMR expressions in 20 cancer tissues. a Stacked bar plot to visualize the degree of OSMR gene expression based on immunohistochemical staining in 20 cancer types. b Immunohistochemical staining of OSMR protein in 20 cancers. The patient IDs, genders, ages as well as the cancer types are shown. Fig. S3 Estimation of OSM-related tumor purity (demonstrated by ESTIMATE Scores) in the tumor microenvironments across cancers in TGCA via ESTIMATE algorithm. Spearman’s p values and partial correlation values are displayed. Fig. S4 Estimation of OSM-related stromal cell infiltration (demonstrated by Stromal Scores) in the tumor microenvironments across cancers in TGCA via ESTIMATE algorithm. Spearman’s p values and partial correlation values are displayed. Fig. S5 Estimation of OSM-related immune cell infiltration (demonstrated by Immune Scores) in the tumor microenvironments across cancers in TGCA via ESTIMATE algorithm. Spearman’s p values and partial correlation values are displayed. Fig. S6 Co-expression analysis of specific molecules (growth factors and cytokines) and OSM of cancers in TCGA. a Heatmap of OSM-related growth factor co-expression analysis. b Heatmap of OSM-related cytokine co-expression analysis. The colors in the bottom right corner of each grid represent Spearman’s correlation values. And the colors in the upper left corner of each grid represent Spearman’s p values. * p < 0.05, ** p < 0.01, *** p < 0.001. Fig. S7 Main OSM-related signaling pathways in tumor microenvironment via GO enrichment analysis. a Histogram of main OSM-up-regulated pathways in the OSM high expression group based on GO enrichment analysis. b Grouped network plot to visualize the enrich [file 12935_2021_2260_MOESM1_ESM.pdf]

Fig. S1

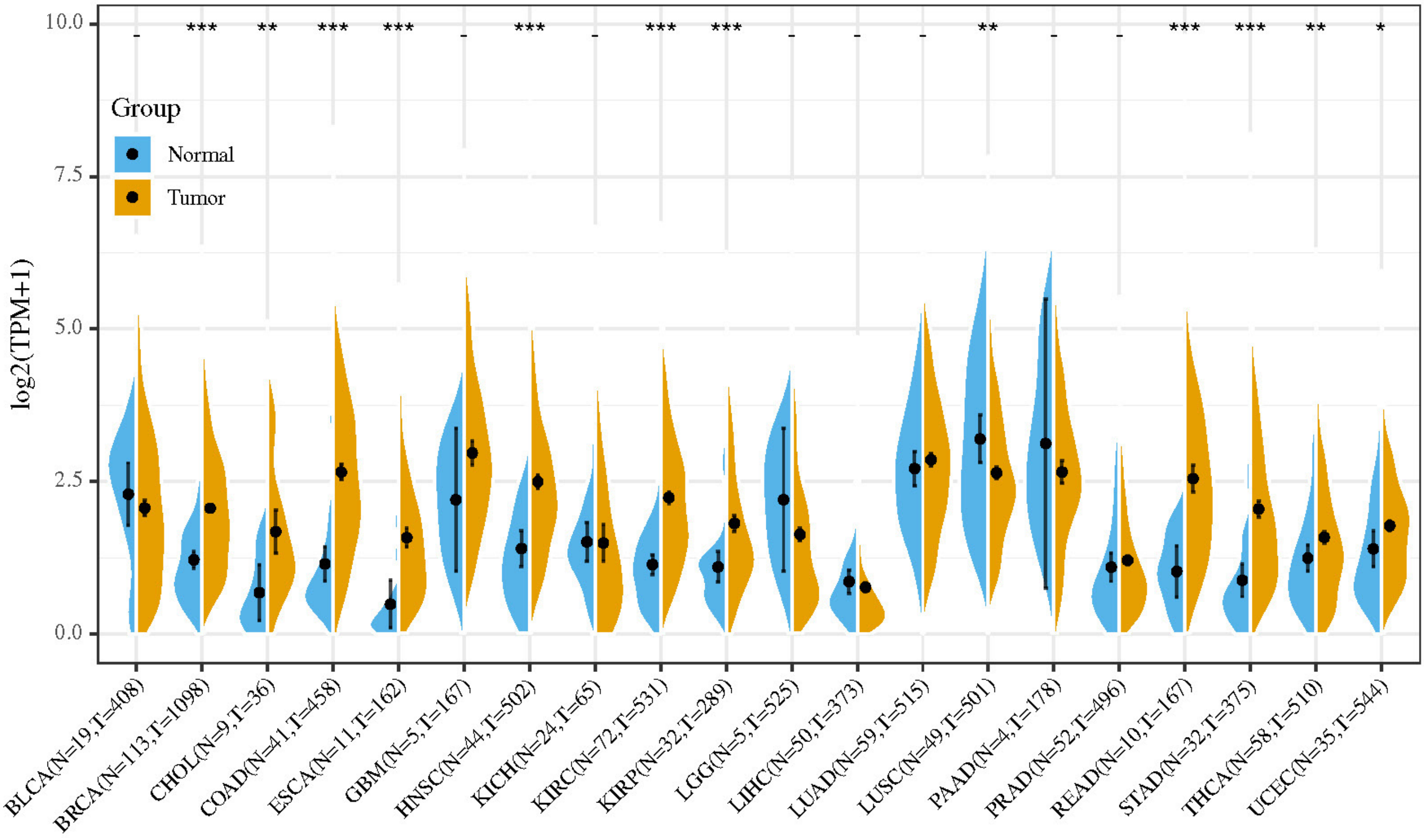
log2(TPM+1)
10.0
7.5
5.0
2.5
0.0
Group
Normal
Tumor
BLCA(N=19,T=408)
BRCA(N=113,T=1098)
CHOL(N=9,T=36)
COAD(N=41,T=458)
ESCA(N=11,T=162)
GBM(N=5,T=167)
HNSC(N=44,T=502)
KICH(N=24,T=65)
KIRC(N=72,T=531)
KIRP(N=32,T=289)
LGG(N=5,T=525)
LIHC(N=50,T=373)
LUAD(N=59,T=515)
LUSC(N=49,T=501)
PAAD(N=4,T=178)
PRAD(N=52,T=496)
READ(N=10,T=167)
STAD(N=32,T=375)
THCA(N=58,T=510)
UCEC(N=35,T=544)

Fig. S2

a

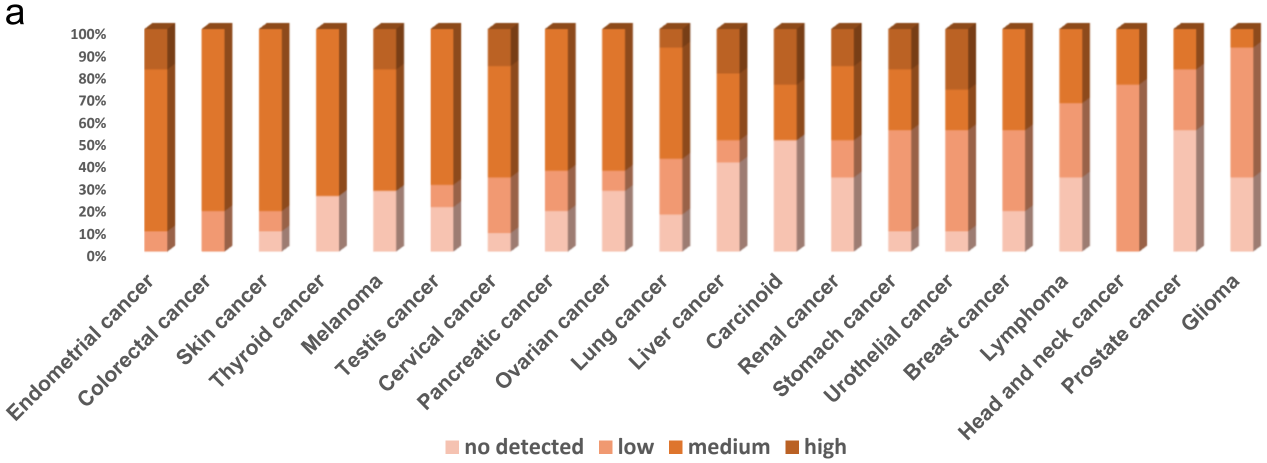
100%
90%
80%
70%
60%
50%
40%
30%
20%
10%
0%
Endometrial cancer
Colorectal cancer
Skin cancer
Thyroid cancer
Melanoma
Testis cancer
Cervical cancer
Pancreatic cancer
Ovarian cancer
Lung cancer
Liver cancer
Carcinoid
Renal cancer
Stomach cancer
Urothelial cancer
Breast cancer
Lymphoma
Head and neck cancer
Prostate cancer
Glioma
no detected
low
medium
high

b

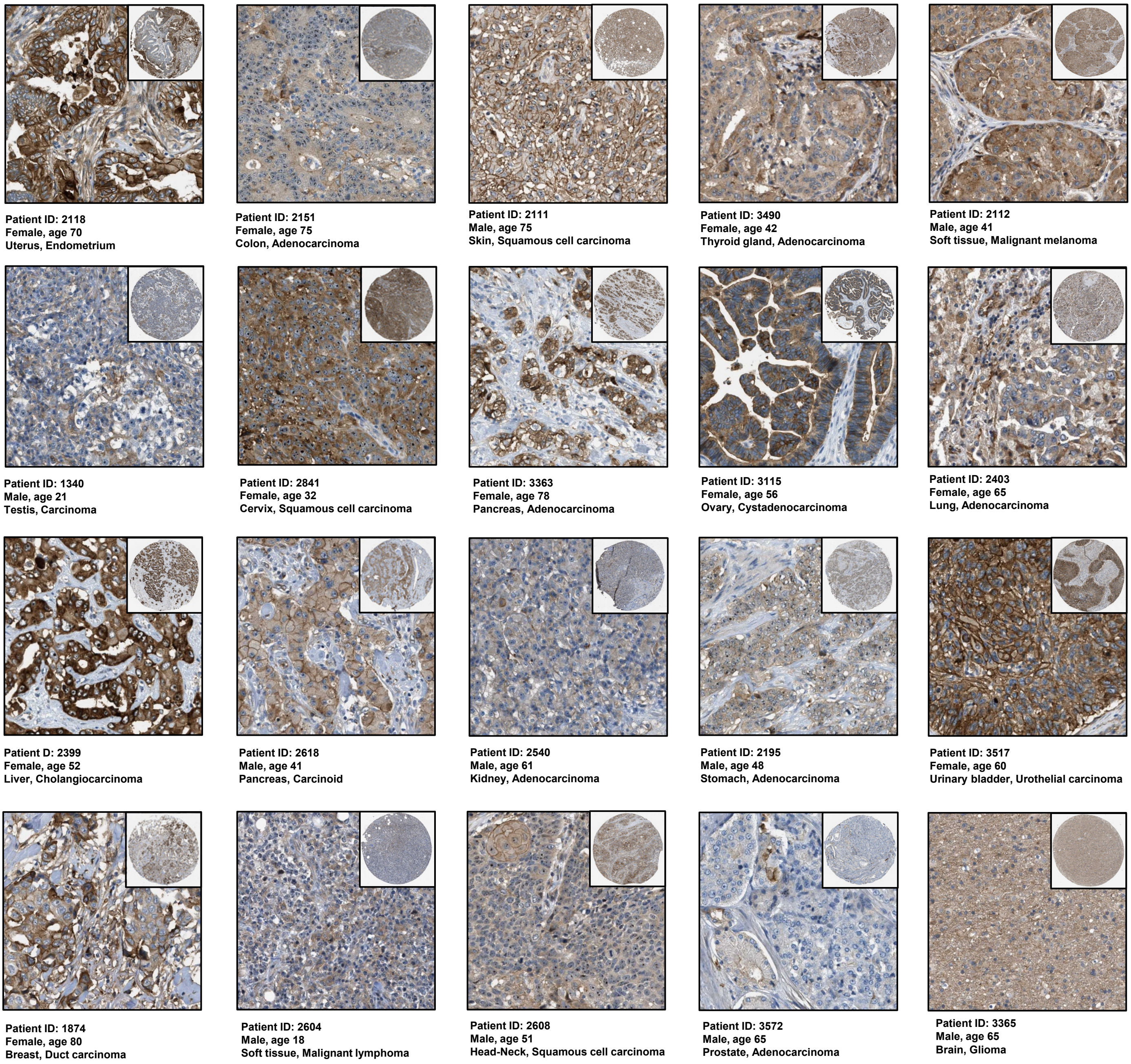
Patient ID: 2118
Female, age 70
Uterus, Endometrium
Patient ID: 2151
Female, age 75
Colon, Adenocarcinoma
Patient ID: 2111
Male, age 75
Skin, Squamous cell carcinoma
Patient ID: 3490
Female, age 42
Thyroid gland, Adenocarcinoma
Patient ID: 2112
Male, age 41
Soft tissue, Malignant melanoma
Patient ID: 1340
Male, age 21
Testis, Carcinoma
Patient ID: 2841
Female, age 32
Cervix, Squamous cell carcinoma
Patient ID: 3363
Female, age 78
Pancreas, Adenocarcinoma
Patient ID: 3115
Female, age 56
Ovary, Cystadenocarcinoma
Patient ID: 2403
Female, age 65
Lung, Adenocarcinoma
Patient D: 2399
Female, age 52
Liver, Cholangiocarcinoma
Patient ID: 2618
Male, age 41
Pancreas, Carcinoid
Patient ID: 2540
Male, age 61
Kidney, Adenocarcinoma
Patient ID: 2195
Male, age 48
Stomach, Adenocarcinoma
Patient ID: 3517
Female, age 60
Urinary bladder, Urothelial carcinoma
Patient ID: 1874
Female, age 80
Breast, Duct carcinoma
Patient ID: 2604
Male, age 18
Soft tissue, Malignant lymphoma
Patient ID: 2608
Male, age 51
Head-Neck, Squamous cell carcinoma
Patient ID: 3572
Male, age 65
Prostate, Adenocarcinoma
Patient ID: 3365
Male, age 65
Brain, Glioma

Fig. S3

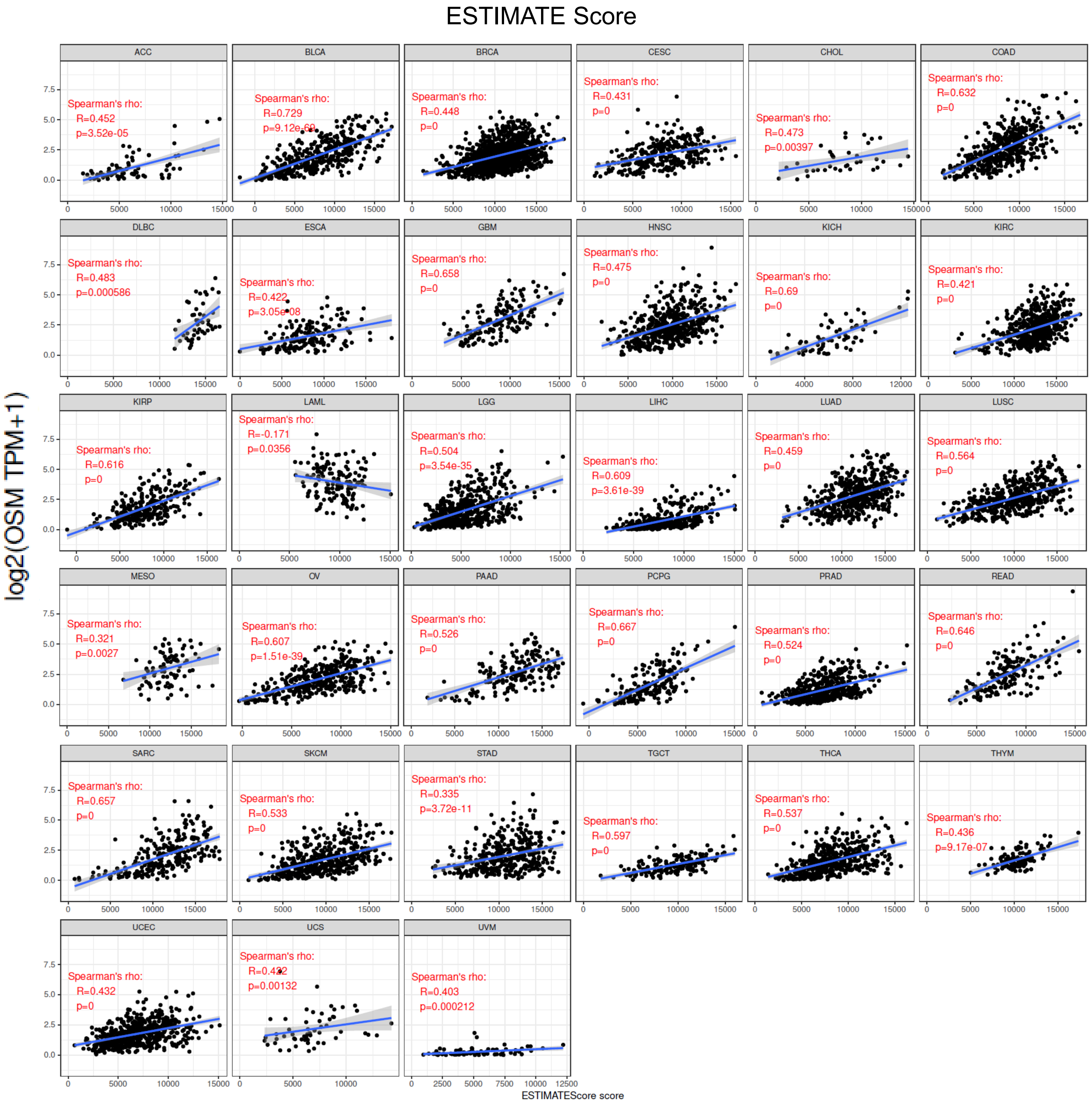
ESTIMATE Score
log2(OSM TPM+1)
ESTIMATEScore score
ACC Spearman's rho: R=0.452 p=3.52e-05
BLCA Spearman's rho: R=0.729 p=9.12e-69
BRCA Spearman's rho: R=0.448 p=0
CESC Spearman's rho: R=0.431 p=0
CHOL Spearman's rho: R=0.473 p=0.00397
COAD Spearman's rho: R=0.632 p=0
DLBC Spearman's rho: R=0.483 p=0.000586
ESCA Spearman's rho: R=0.422 p=3.05e-08
GBM Spearman's rho: R=0.658 p=0
HNSC Spearman's rho: R=0.475 p=0
KICH Spearman's rho: R=0.69 p=0
KIRC Spearman's rho: R=0.421 p=0
KIRP Spearman's rho: R=0.616 p=0
LAML Spearman's rho: R=-0.171 p=0.0356
LGG Spearman's rho: R=0.504 p=3.54e-35
LIHC Spearman's rho: R=0.609 p=3.61e-39
LUAD Spearman's rho: R=0.459 p=0
LUSC Spearman's rho: R=0.564 p=0
MESO Spearman's rho: R=0.321 p=0.0027
OV Spearman's rho: R=0.607 p=1.51e-39
PAAD Spearman's rho: R=0.526 p=0
PCPG Spearman's rho: R=0.667 p=0
PRAD Spearman's rho: R=0.524 p=0
READ Spearman's rho: R=0.646 p=0
SARC Spearman's rho: R=0.657 p=0
SKCM Spearman's rho: R=0.533 p=0
STAD Spearman's rho: R=0.335 p=3.72e-11
TGCT Spearman's rho: R=0.597 p=0
THCA Spearman's rho: R=0.537 p=0
THYM Spearman's rho: R=0.436 p=9.17e-07
UCEC Spearman's rho: R=0.432 p=0
UCS Spearman's rho: R=0.432 p=0.00132
UVM Spearman's rho: R=0.403 p=0.000212

Fig. S4

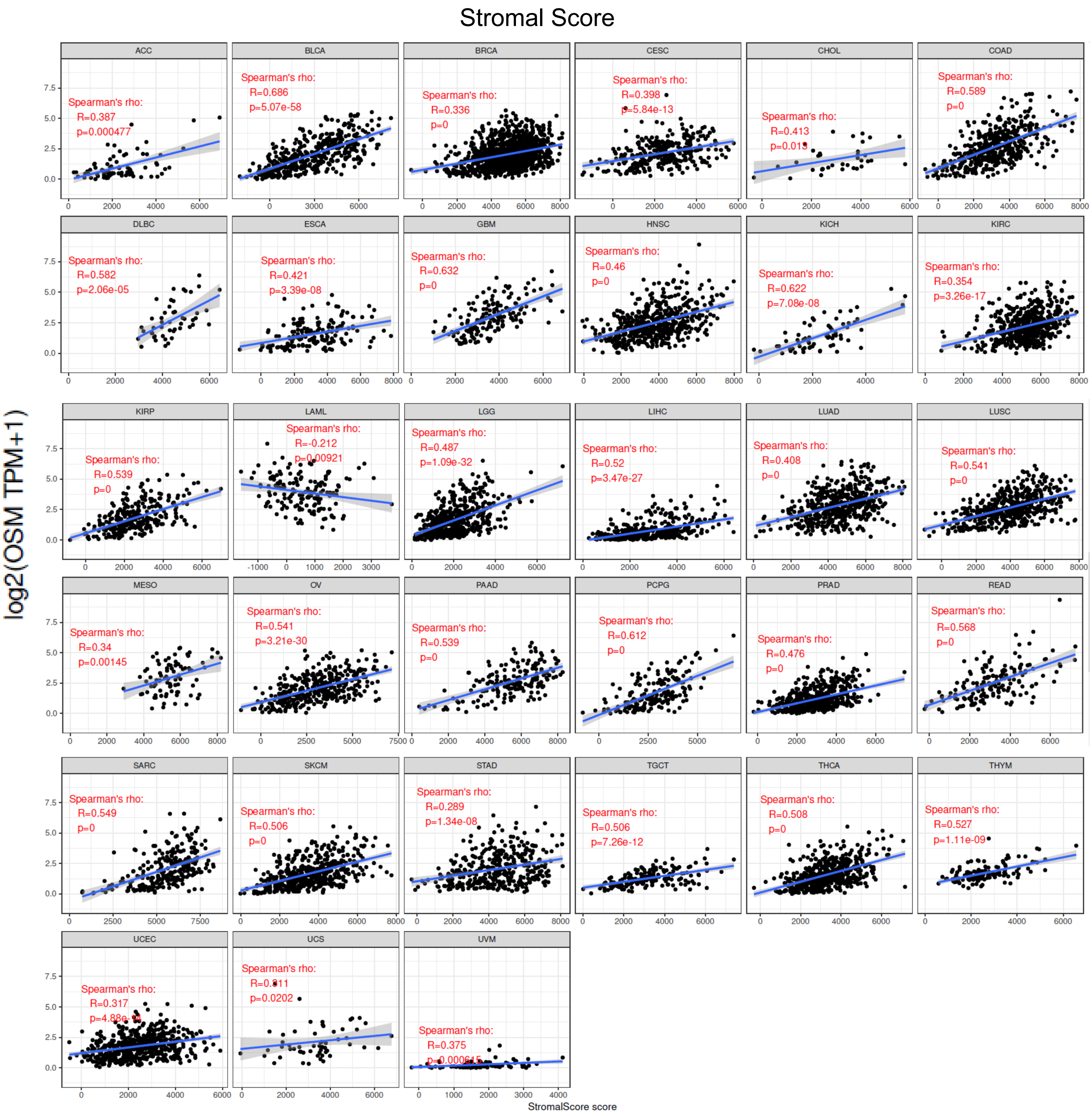
Stromal Score
log2(OSM TPM+1)
StromalScore score
ACC
Spearman's rho: R=0.387 p=0.000477
BLCA
Spearman's rho: R=0.686 p=5.07e-58
BRCA
Spearman's rho: R=0.336 p=0
CESC
Spearman's rho: R=0.398 p=5.84e-13
CHOL
Spearman's rho: R=0.413 p=0.013
COAD
Spearman's rho: R=0.589 p=0
DLBC
Spearman's rho: R=0.582 p=2.06e-05
ESCA
Spearman's rho: R=0.421 p=3.39e-08
GBM
Spearman's rho: R=0.632 p=0
HNSC
Spearman's rho: R=0.46 p=0
KICH
Spearman's rho: R=0.622 p=7.08e-08
KIRC
Spearman's rho: R=0.354 p=3.26e-17
KIRP
Spearman's rho: R=0.539 p=0
LAML
Spearman's rho: R=-0.212 p=0.00921
LGG
Spearman's rho: R=0.487 p=1.09e-32
LIHC
Spearman's rho: R=0.52 p=3.47e-27
LUAD
Spearman's rho: R=0.408 p=0
LUSC
Spearman's rho: R=0.541 p=0
MESO
Spearman's rho: R=0.34 p=0.00145
OV
Spearman's rho: R=0.541 p=3.21e-30
PAAD
Spearman's rho: R=0.539 p=0
PCPG
Spearman's rho: R=0.612 p=0
PRAD
Spearman's rho: R=0.476 p=0
READ
Spearman's rho: R=0.568 p=0
SARC
Spearman's rho: R=0.549 p=0
SKCM
Spearman's rho: R=0.506 p=0
STAD
Spearman's rho: R=0.289 p=1.34e-08
TGCT
Spearman's rho: R=0.506 p=7.26e-12
THCA
Spearman's rho: R=0.508 p=0
THYM
Spearman's rho: R=0.527 p=1.11e-09
UCEC
Spearman's rho: R=0.317
UCS
Spearman's rho: p=0.0202
UVM
Spearman's rho: R=0.375 p=0.000615

Fig. S5

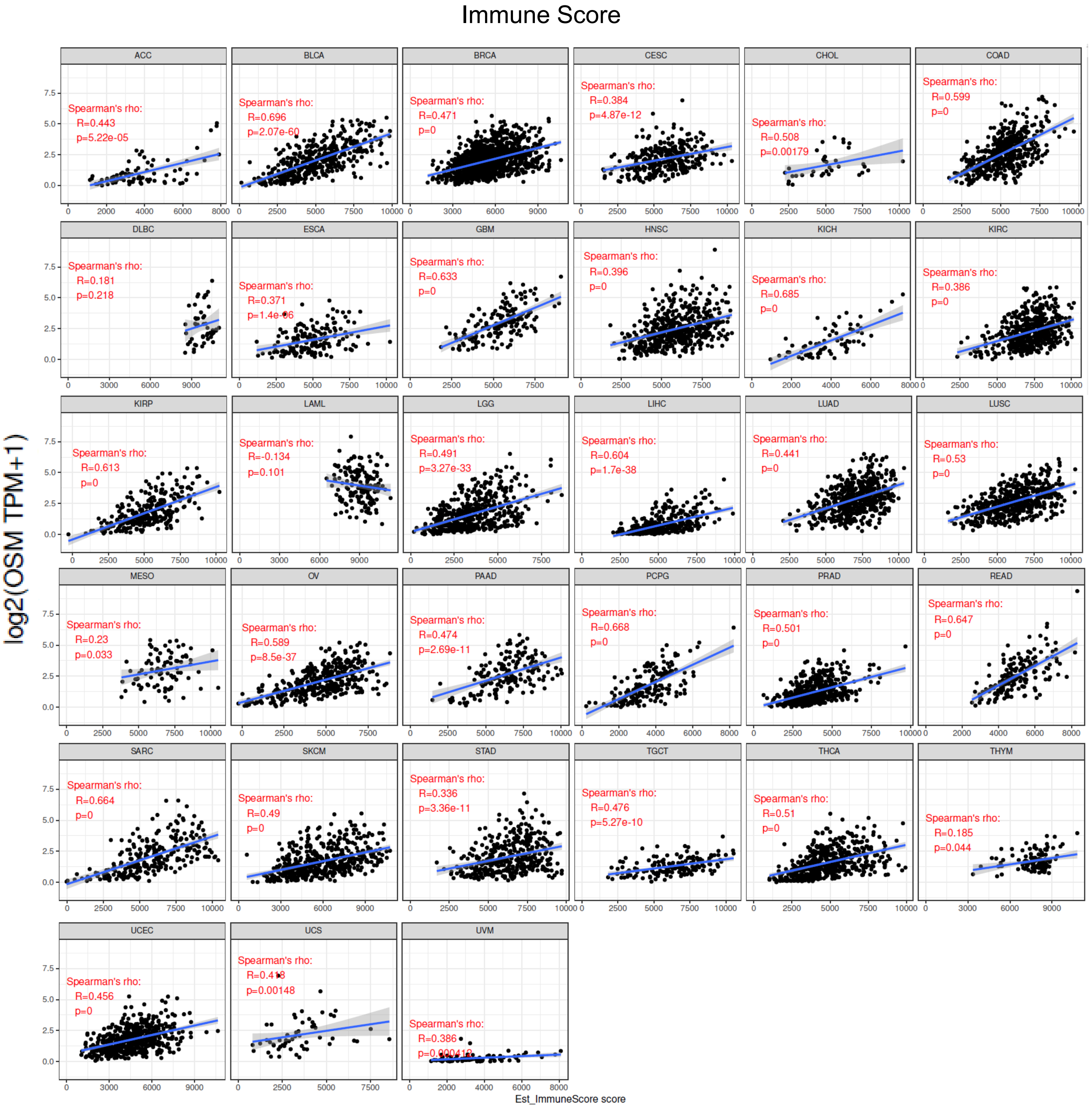
Immune Score
log2(OSM TPM+1)
Est_ImmuneScore score
ACC
Spearman's rho:
R=0.443
p=5.22e-05
BLCA
Spearman's rho:
R=0.696
p=2.07e-60
BRCA
Spearman's rho:
R=0.471
p=0
CESC
Spearman's rho:
R=0.384
p=4.87e-12
CHOL
Spearman's rho:
R=0.508
p=0.00179
COAD
Spearman's rho:
R=0.599
p=0
DLBC
Spearman's rho:
R=0.181
p=0.218
ESCA
Spearman's rho:
R=0.371
p=1.4e-06
GBM
Spearman's rho:
R=0.633
p=0
HNSC
Spearman's rho:
R=0.396
p=0
KICH
Spearman's rho:
R=0.685
p=0
KIRC
Spearman's rho:
R=0.386
p=0
KIRP
Spearman's rho:
R=0.613
p=0
LAML
Spearman's rho:
R=-0.134
p=0.101
LGG
Spearman's rho:
R=0.491
p=3.27e-33
LIHC
Spearman's rho:
R=0.604
p=1.7e-38
LUAD
Spearman's rho:
R=0.441
p=0
LUSC
Spearman's rho:
R=0.53
p=0
MESO
Spearman's rho:
R=0.23
p=0.033
OV
Spearman's rho:
R=0.589
p=8.5e-37
PAAD
Spearman's rho:
R=0.474
p=2.69e-11
PCPG
Spearman's rho:
R=0.668
p=0
PRAD
Spearman's rho:
R=0.501
p=0
READ
Spearman's rho:
R=0.647
p=0
SARC
Spearman's rho:
R=0.664
p=0
SKCM
Spearman's rho:
R=0.49
p=0
STAD
Spearman's rho:
R=0.336
p=3.36e-11
TGCT
Spearman's rho:
R=0.476
p=5.27e-10
THCA
Spearman's rho:
R=0.51
p=0
THYM
Spearman's rho:
R=0.185
p=0.044
UCEC
Spearman's rho:
R=0.456
p=0
UCS
Spearman's rho:
R=0.418
p=0.00148
UVM
Spearman's rho:
R=0.386

Fig. S6

a

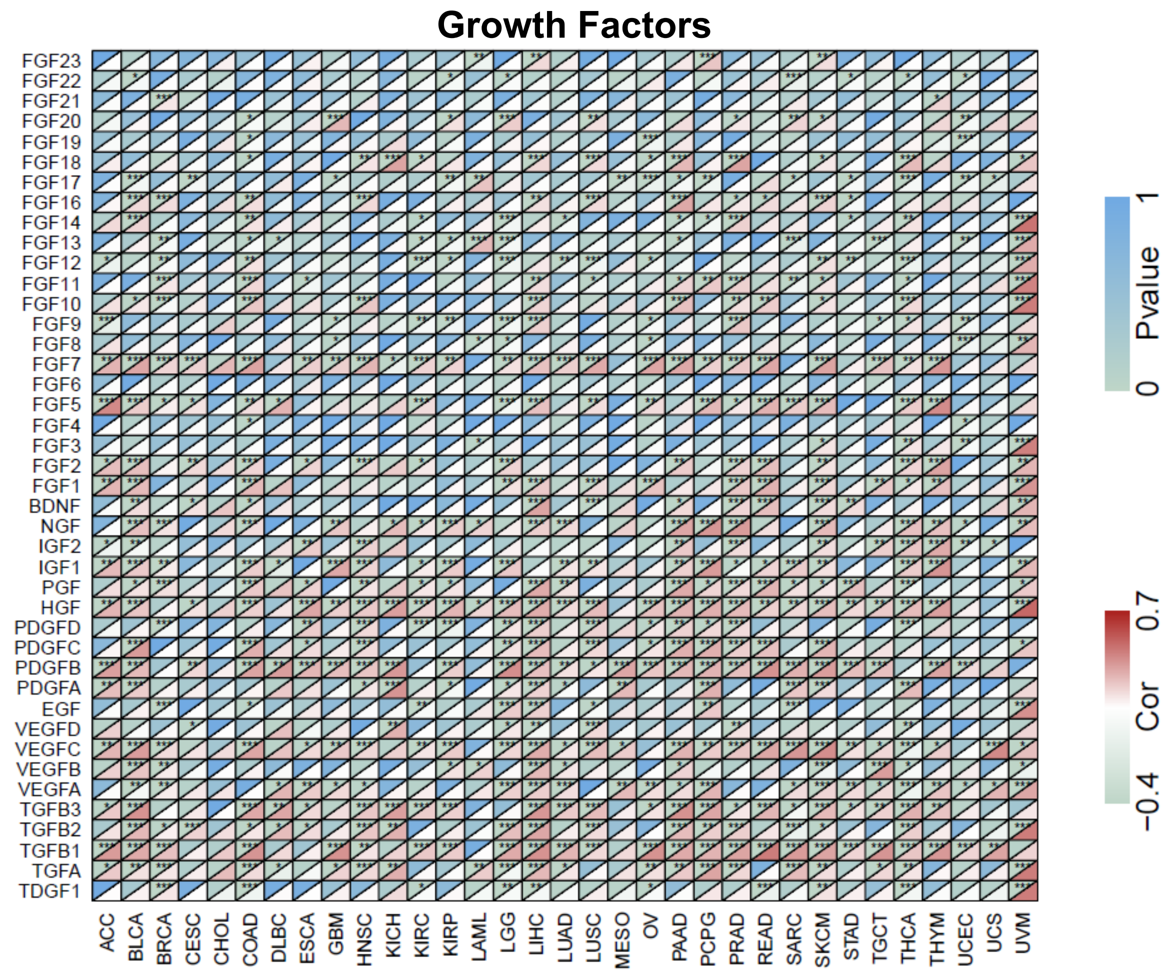
Growth Factors
FGF23
FGF22
FGF21
FGF20
FGF19
FGF18
FGF17
FGF16
FGF14
FGF13
FGF12
FGF11
FGF10
FGF9
FGF8
FGF7
FGF6
FGF5
FGF4
FGF3
FGF2
FGF1
BDNF
NGF
IGF2
IGF1
PGF
HGF
PDGFD
PDGFC
PDGFB
PDGFA
EGF
VEGFD
VEGFC
VEGFB
VEGFA
TGFB3
TGFB2
TGFB1
TGFA
TDGF1
ACC BLCA BRCA CESC CHOL COAD DLBC ESCA GBM HNSC KICH KIRC KIRP LAML LGG LIHC LUAD LUSC MESO OV PAAD PCPG PRAD READ SARC SKCM STAD TGCT THCA THYM UCEC UCS UVM
Pvalue
1
0
Cor
0.7
−0.4

b

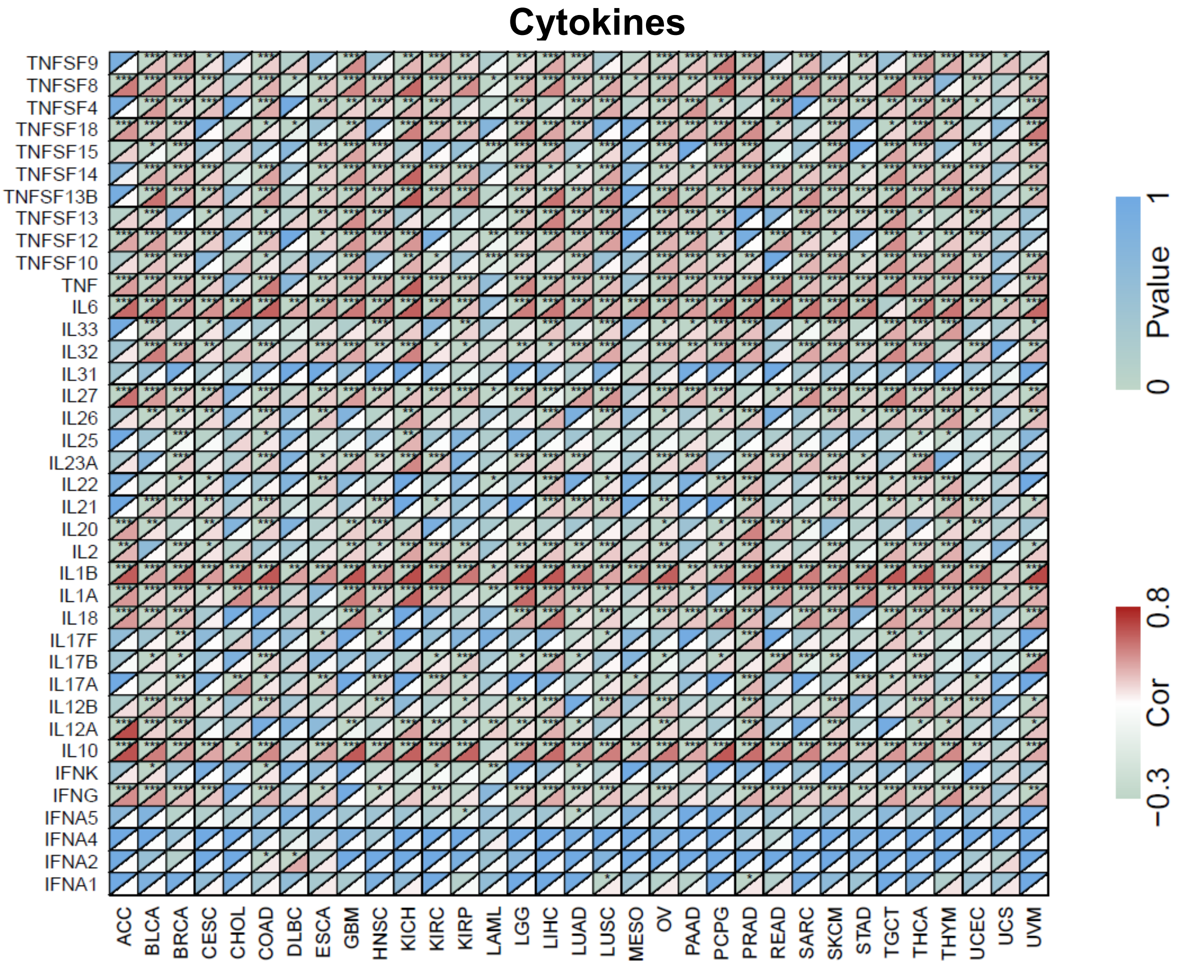
Cytokines
TNFSF9
TNFSF8
TNFSF4
TNFSF18
TNFSF15
TNFSF14
TNFSF13B
TNFSF13
TNFSF12
TNFSF10
TNF
IL6
IL33
IL32
IL31
IL27
IL26
IL25
IL23A
IL22
IL21
IL20
IL2
IL1B
IL1A
IL18
IL17F
IL17B
IL17A
IL12B
IL12A
IL10
IFNK
IFNG
IFNA5
IFNA4
IFNA2
IFNA1
ACC BLCA BRCA CESC CHOL COAD DLBC ESCA GBM HNSC KICH KIRC KIRP LAML LGG LIHC LUAD LUSC MESO OV PAAD PCPG PRAD READ SARC SKCM STAD TGCT THCA THYM UCEC UCS UVM
Pvalue
1
0
Cor
0.8
−0.3

Fig. S7

a

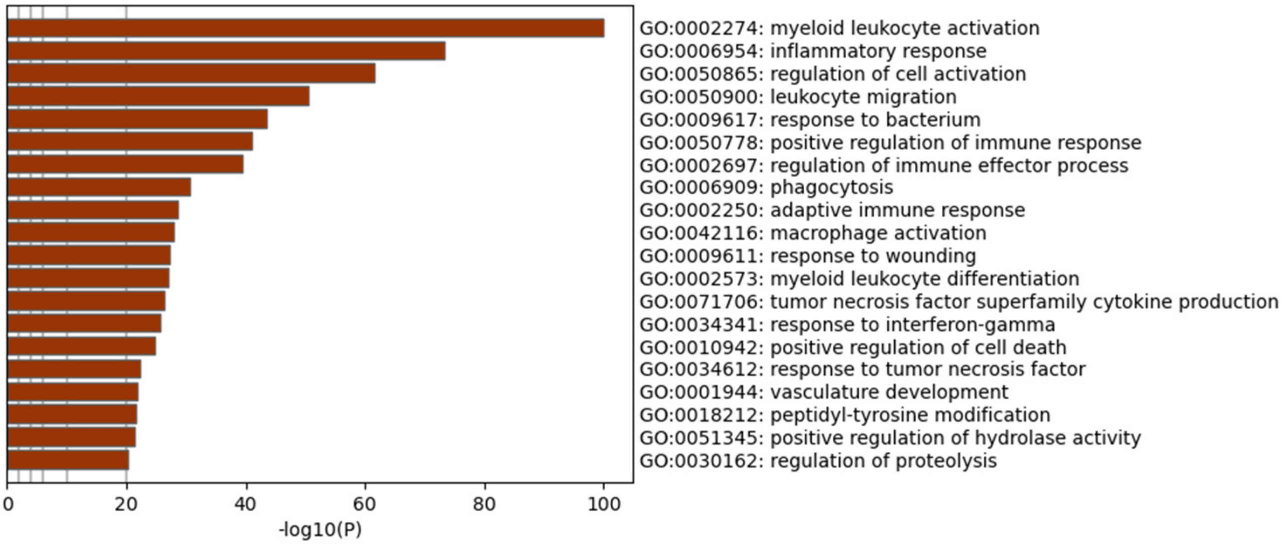
GO:0002274: myeloid leukocyte activation
GO:0006954: inflammatory response
GO:0050865: regulation of cell activation
GO:0050900: leukocyte migration
GO:0009617: response to bacterium
GO:0050778: positive regulation of immune response
GO:0002697: regulation of immune effector process
GO:0006909: phagocytosis
GO:0002250: adaptive immune response
GO:0042116: macrophage activation
GO:0009611: response to wounding
GO:0002573: myeloid leukocyte differentiation
GO:0071706: tumor necrosis factor superfamily cytokine production
GO:0034341: response to interferon-gamma
GO:0010942: positive regulation of cell death
GO:0034612: response to tumor necrosis factor
GO:0001944: vasculature development
GO:0018212: peptidyl-tyrosine modification
GO:0051345: positive regulation of hydrolase activity
GO:0030162: regulation of proteolysis
0
20
40
60
80
100
-log10(P)

b

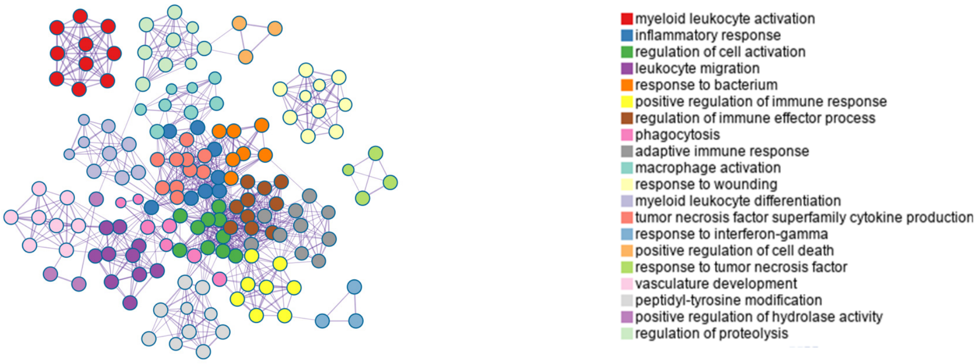
myeloid leukocyte activation
inflammatory response
regulation of cell activation
leukocyte migration
response to bacterium
positive regulation of immune response
regulation of immune effector process
phagocytosis
adaptive immune response
macrophage activation
response to wounding
myeloid leukocyte differentiation
tumor necrosis factor superfamily cytokine production
response to interferon-gamma
positive regulation of cell death
response to tumor necrosis factor
vasculature development
peptidyl-tyrosine modification
positive regulation of hydrolase activity
regulation of proteolysis

c

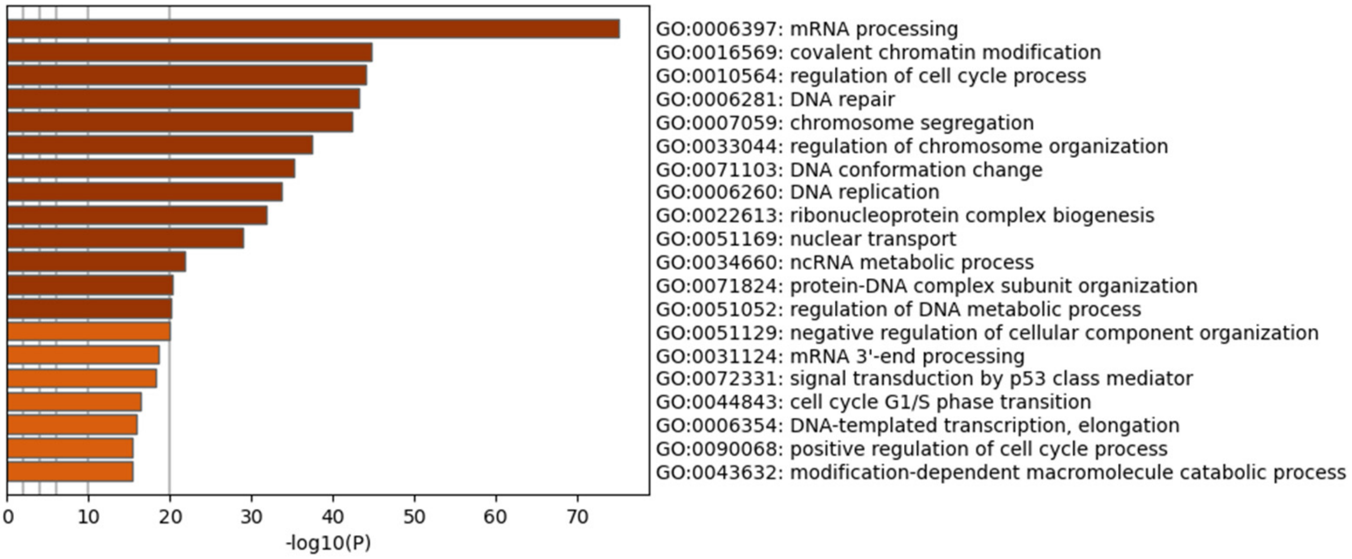
GO:0006397: mRNA processing
GO:0016569: covalent chromatin modification
GO:0010564: regulation of cell cycle process
GO:0006281: DNA repair
GO:0007059: chromosome segregation
GO:0033044: regulation of chromosome organization
GO:0071103: DNA conformation change
GO:0006260: DNA replication
GO:0022613: ribonucleoprotein complex biogenesis
GO:0051169: nuclear transport
GO:0034660: ncRNA metabolic process
GO:0071824: protein-DNA complex subunit organization
GO:0051052: regulation of DNA metabolic process
GO:0051129: negative regulation of cellular component organization
GO:0031124: mRNA 3'-end processing
GO:0072331: signal transduction by p53 class mediator
GO:0044843: cell cycle G1/S phase transition
GO:0006354: DNA-templated transcription, elongation
GO:0090068: positive regulation of cell cycle process
GO:0043632: modification-dependent macromolecule catabolic process
0
10
20
30
40
50
60
70
-log10(P)

d

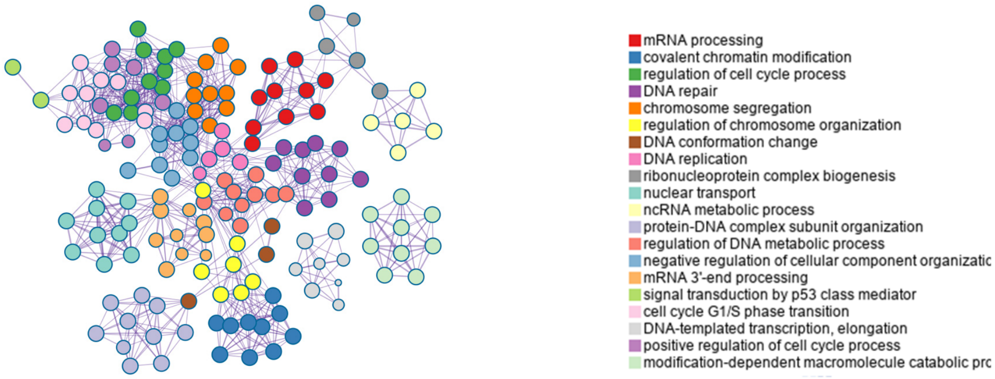
mRNA processing
covalent chromatin modification
regulation of cell cycle process
DNA repair
chromosome segregation
regulation of chromosome organization
DNA conformation change
DNA replication
ribonucleoprotein complex biogenesis
nuclear transport
ncRNA metabolic process
protein-DNA complex subunit organization
regulation of DNA metabolic process
negative regulation of cellular component organizatic
mRNA 3'-end processing
signal transduction by p53 class mediator
cell cycle G1/S phase transition
DNA-templated transcription, elongation
positive regulation of cell cycle process
modification-dependent macromolecule catabolic prc

Fig. S8

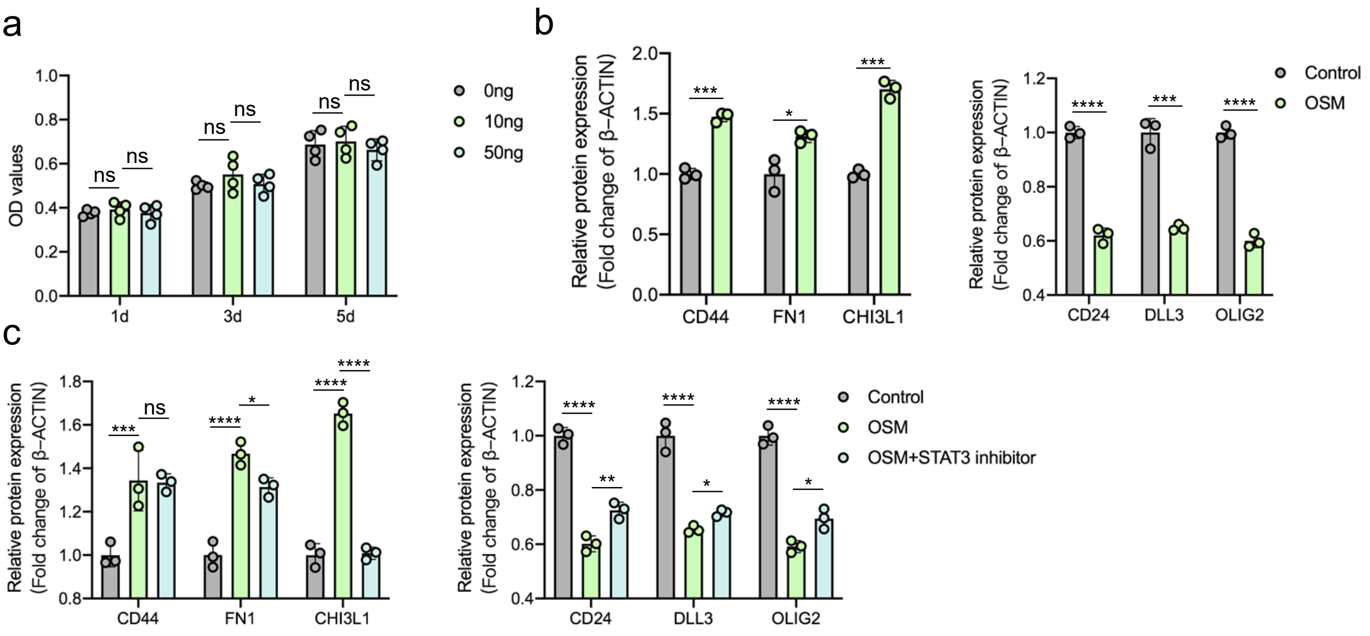
a
OD values
1d
3d
5d
ns
0ng
10ng
50ng
b
Relative protein expression
(Fold change of β−ACTIN)
CD44
FN1
CHI3L1
CD24
DLL3
OLIG2
Control
OSM
c
OSM+STAT3 inhibitor
